# Exploring the Biological Activity of Phytocannabinoid Formulations for Skin Health Care: A Special Focus on Molecular Pathways

**DOI:** 10.3390/ijms252313142

**Published:** 2024-12-06

**Authors:** Guilherme Trigo, Mariana Coelho, Carolina Borges Ferreira, Matteo Melosini, Inês Sousa Lehmann, Catarina P. Reis, Maria Manuela Gaspar, Susana Santos

**Affiliations:** 1 R&D&I Department, EXMceuticals Portugal Lda, 1749-016 Lisboa, Portugal; guilherme@tamartech.com (G.T.); carolina@tamartech.com (C.B.F.); matteo@tamartech.com (M.M.); ines@tamartech.com (I.S.L.); susana@tamartech.com (S.S.); 2 Research Institute for Medicines (iMed.ULisboa), Faculty of Pharmacy, Universidade de Lisboa, Avenida Professor Gama Pinto, 1649-003 Lisboa, Portugal; mariana.coelho@ff.ulisboa.pt; 3 Institute of Biophysics and Biomedical Engineering (IBEB), Faculty of Sciences, Universidade de Lisboa, Campo Grande, 1749-016 Lisboa, Portugal

**Keywords:** endocannabinoid system (ECS), skin, inflammation, regeneration, phytocannabinoids (pCBs), cosmeceutical applications, therapeutics

## Abstract

Recent advancements have highlighted the potential of cannabis and its phytocannabinoids (pCBs) in skin health applications. These compounds, through their interaction with the endocannabinoid system (ECS), show promise for skin health products. Their ability to regulate inflammation, oxidative stress and cell proliferation makes them useful in addressing skin problems such as inflammation, scarring, healing, acne and aging, positioning them as valuable tools for innovative skincare solutions. In the present work, the cellular and molecular effects of proprietary pCB-based formulations on ECS modulation, inflammation and skin regeneration were investigated. Using human dermal fibroblasts (HDF) and keratinocytes (HaCaT), the effect of formulations in both pre-treatment and treatment scenarios following exposure to stress-inducing agents was assessed. Key molecular markers were analyzed to tackle their efficacy in mitigating inflammation and promoting structural integrity and regeneration. In vitro results showed that these formulations significantly reduced inflammation, promoted skin regeneration and improved structural functions. In vivo studies confirmed that the formulations were well-tolerated and led to noticeable improvements in skin health, including enhanced barrier function. This study demonstrates the safety and efficacy of pCB-based formulations for cosmeceutical applications. By combining molecular analysis with in vivo testing, this research provides new insights into the therapeutic potential of pCBs for managing various skin conditions.

## 1. Introduction

*Cannabis sativa* is a complex plant with a long and outstanding history spanning over 4000 years, particularly in Asia, with significant use in India for religious, recreational and medicinal purposes [1]. In recent years, there has been an increased interest by the scientific community in cannabis and its constituents, aiming to explore their therapeutic potential [2]. The diverse phytochemical profile of *Cannabis sativa* contributes to its wide-ranging effects and potential medical applications. The plant contains over 500 distinct compounds, including terpenes, flavonoids, alkaloids and cannabinoids [3]. To date, approximately 125 cannabinoids have been identified and characterized [1], and these bioactive compounds are generally classified based on their origin [4,5]. The three primary classes of cannabinoids are as follows:

- Endocannabinoids, which are naturally synthesized in the human body. This class includes anandamide (EAE) and 2-arachidonoylglycerol (2-AG), which interact with the endocannabinoid system (ECS).
- Phytocannabinoids (pCBs), which are produced by plants. Among the most studied pCBs are Δ9-tetrahydrocannabinol (THC) and cannabidiol (CBD), though many others have been identified and are currently under investigation for their potential therapeutic effects.
- Synthetic cannabinoids, which are artificially synthesized in laboratories [4,5].

Our research specifically focused on pCBs from *Cannabis sativa*. To date, more than 120 distinct pCBs have been identified, reflecting the plant’s remarkable chemical diversity [6]. These pCBs interact with the human ECS, influencing various physiological processes.

The unique properties of different pCBs have led to intense research in areas such as pain management and neurological disorders. In dermatology, the most studied pCBs include CBD, cannabigerol (CBG), cannabichromene (CBC), cannabinol (CBN) and THC [4,5]. These compounds have been explored for their potential roles in modulating skin homeostasis, including effects on inflammation, cell proliferation, sebum production, skin barrier function and wound healing.

In vitro studies have provided substantial evidence for the pharmacological effects of these pCBs on various skin-related cell lines, including keratinocytes, sebocytes and fibroblasts [4]. For instance, CBD has been shown to inhibit the proliferation of keratinocytes, suggesting its potential role in managing hyperproliferative skin disorders like psoriasis [5]. Similarly, CBG has been found to induce apoptosis in human skin fibroblasts, highlighting its potential utility in managing fibrotic skin diseases [6]. These findings underscore the therapeutic potential of pCBs in skin disorders, warranting further investigation into their mechanisms of action and their applications in both cosmeceutical and clinical settings.

The ECS is a complex signaling system present in both the central and peripheral nervous systems, as well as in many peripheral tissues [7,8]. It comprises cannabinoid receptors (CB1R and CB2R), endocannabinoids such as AEA and 2-AG, and the enzymes responsible for their synthesis and degradation [9,10]. Initially discovered in the brain, the ECS has since been found to extend its regulatory functions to various peripheral tissues, influencing numerous physiological pathways, including pain perception, gastrointestinal activity, hormonal regulation, cardiovascular function, immune system modulation and inflammatory responses [11,12].

The ECS plays a crucial role in maintaining skin homeostasis, regulating inflammation, and promoting skin regeneration by influencing various physiological processes, including cell proliferation, differentiation and apoptosis. The interaction between endocannabinoids and their receptors in the skin suggests a significant role in maintaining skin health and responding to stressors. Recent scientific advancements have highlighted the potential therapeutic applications of cannabinoids in dermatological formulations, particularly in addressing skin disorders through the modulation of the ECS.

pCBs such as CBD, CBG and CBC have garnered attention for their potential benefits in cosmeceutical formulations. Importantly, pCBs have different affinities towards human receptors, and the biological effects are often the result of the interaction with multiple molecular targets [4,13,14,15].

These compounds interact with ECS receptors in the skin, offering anti-inflammatory and antioxidant properties, and hence the topical application of pCBs can potentially address various skin disorders, including eczema, psoriasis and acne, by modulating inflammatory pathways and promoting skin regeneration [16,17,18].

CBD, one of the most studied non-psychoactive pCBs, has been shown to possess anti-inflammatory, antioxidant, and anti-proliferative properties. It modulates skin homeostasis by influencing key signaling pathways involved in skin cell proliferation and differentiation. Also, it has demonstrated efficacy in regulating the production of sebum in sebocytes and in reducing inflammation in keratinocytes, making it a promising candidate for the treatment of acne vulgaris and other inflammatory skin conditions [19,20]. Previous studies reported that in keratinocytes, the pCBs, CBD, CBG, and CBC, were able to reduce the inflammation [21,22]. Sangiovanni and colleagues evaluated the effect of CBD on the wound healing and inflammation processes, using TNF-α as a pro-inflammatory stimulus. They found that CBD not only protected the cells but also induced the expression of genes related to antioxidant and detoxification systems, as well as those involved in keratinocyte differentiation and skin development [23]. In human fibroblasts, CBD was able to reduce inflammation and regulate the extracellular matrix (ECM). The authors observed a decrease in the cytokines’ expression and an activation of proteinases that regulate the ECM components [21,24]. Atalay et al. (2020) investigated the antioxidant properties of CBD in keratinocytes exposed to UVB radiation and hydrogen peroxide (H_2_O_2_). The study found that CBD upregulated the expression of several antioxidant genes, including superoxide dismutase and catalase. This upregulation provided significant protection against oxidative damage, suggesting that CBD could be used to prevent or mitigate oxidative stress-related skin aging and damage [25]. A study by Palmieri et al. (2019) evaluated the effects of a CBD-enriched ointment on inflammatory skin diseases, demonstrating improvements in various clinical parameters and quality of life measures [26].

CBG, although less abundant in the cannabis plant, has also shown potential benefits for skin care. Research indicates that CBG may have antimicrobial, anti-inflammatory, and analgesic properties, which could be beneficial in treating skin conditions characterized by inflammation and pain. CBG has been observed to influence the ECS in the skin, particularly through its interaction with cannabinoid receptors, potentially modulating inflammatory responses and skin cell proliferation [27,28]. Pérez et al. (2022) conducted in vitro and clinical evaluations of CBG using gene expression assays. The study demonstrated that CBG has a broad range of anti-inflammatory effects, modulating the expression of various genes involved in skin inflammation and immune responses. This work expands the scope of cannabinoids studied for dermatological applications beyond CBD, indicating that CBG could also play a significant role in skin health [17].

CBC has demonstrated potential as an anti-inflammatory and antimicrobial agent. Its ability to inhibit the lipoxygenase enzyme suggests that CBC may play a role in modulating inflammatory processes within the skin. Furthermore, CBC has shown potential in promoting skin homeostasis by influencing the activity of sebaceous glands, thereby offering a possible therapeutic avenue for conditions like acne [27].

In the context of skin physiology, cannabinoid receptors play pivotal roles in maintaining skin homeostasis across various cell types like keratinocytes, fibroblasts, and immune cells. Tóth et al. (2019) underscored the critical influence of CB1 and CB2 receptors on processes such as cell proliferation, differentiation, and immune response regulation in the skin. In dermal fibroblasts, CBD’s interaction with CB1 and CB2 receptors influences collagen production and skin elasticity, suggesting applications in anti-aging and wound healing. Moreover, the expression of structural and repair-associated genes provides insights into the regenerative effects of pCB-based formulations. Key genes like fibronectin (FN1), epidermal growth factor receptor (EGFR), and metallopeptidase inhibitor 3 (TIMP3) are crucial for skin differentiation, barrier function, and tissue repair [29]. Casares et al. (2019) examined CBD’s impact on gene expression using a 3D model of human epidermis, highlighting CBD’s ability to modulate FN1, EGFR, and TIMP3 expression. This modulation promotes skin health by enhancing barrier formation and tissue integrity, emphasizing CBD’s potential therapeutic benefits in skincare formulations [23].

The antioxidant and anti-inflammatory properties of CBD, CBG, and CBC make them highly promising candidates for enhancing overall skin health and promoting the regeneration of damaged skin tissues [17,25,30]. As an example, the anti-inflammatory effects of pCBS are crucial in managing skin disorders characterized by chronic inflammation, such as psoriasis, eczema and acne. In these conditions, inflammation can cause redness, swelling, and discomfort, exacerbating the symptoms and delaying the healing process [31]. pCBs can help reduce this inflammation by modulating the skin’s immune response, leading to calmer, less irritated skin. These compounds possess the ability to neutralize free radicals, which are reactive molecules that cause oxidative stress—a major factor in skin aging and the development of various skin conditions. By mitigating oxidative stress, pCBs help protect the skin from environmental damage, such as UV radiation and pollution, which can lead to premature aging, wrinkles and loss of skin elasticity. By reducing both oxidative and chemical stress, pCBs support the skin’s natural barrier function, helping to maintain hydration and prevent the entry of harmful substances. These properties suggest that pCBs could be valuable components in skincare formulations aimed at improving skin resilience, reducing the signs of aging and treating chronic inflammatory skin diseases.

Considering the significant cosmetic and therapeutic potential of pCBs, the primary objective of the present study was to evaluate, in vitro, the effects of pCBs-based formulations on various human cell lines subjected to stress conditions. To achieve this, we used keratinocytes (HaCaT) and human dermal fibroblasts (HDF). The expression levels of target genes associated with inflammation and regenerative processes were assessed and compared between cells treated with pCBs-based formulations and untreated controls.

The use of HaCaT and HDF cell lines offers valuable insights into inflammation, regeneration and wound healing. Keratinocytes are the predominant cell type in the epidermis and play a key role in the skin’s response to injury and inflammation. Cannabinoid research shows that pCBs enhance keratinocyte migration and modulate cytokine production, exerting anti-inflammatory effects and restoring the skin barrier [1,20]. This migration is a vital step in the re-epithelialization process during wound repair, thus restoring the skin’s barrier function. HDF are essential for studying tissue repair and ECM remodeling, as pCBs promote fibroblast proliferation, migration, and collagen production, supporting wound closure and minimizing scarring by balancing inflammation and regeneration [32].

HaCaT and HDF cells express key proteins like Protein Kinase C (PKC), Nuclear Factor kappa B (NF-κB), and Peroxisome Proliferator-Activated Receptor gamma (PPARγ), crucial for inflammation, stress responses and skin homeostasis. In HaCaT cells, PKC activation, such as by phorbol esters like phorbol 12-myrstate 13-acetate (TPA), can stimulate the NF-κB pathway and activate matrix metalloproteinases (MMPs). The NF-κB pathway, active in both cell types, is a central mediator of inflammatory responses. Upon activation in HaCaT cells, NF-κB translocate to the nucleus, promoting the transcription of pro-inflammatory genes including tumor necrosis factor alpha (TNF-α), interleukin 36G (IL-36G), and interleukin 6 (IL-6). PPARγ is expressed in both HDF and HaCaT cells. While its specific role in these cell lines requires further elucidation, PPARγ is known to be involved in regulating inflammation and maintaining skin homeostasis. MMPs are also expressed in both cell types, with their activation in HaCaT cells linked to PKC stimulation under conditions of chemical stress. pCBs, while not endogenously expressed, exert significant effects on both HDF and HaCaT cells. Recent studies have demonstrated that pCBs modulate inflammatory responses in HaCaT cells [22,33,34,35]. These effects are mediated through the ECS and signaling pathways. It is important to note that while both HDF and HaCaT cells express these proteins and pathways, their expression levels and responses to stimuli may differ due to their distinct origins and functions within the skin. This underscores the importance of considering cell type-specific responses when interpreting results from in vitro studies using these models in skin biology and inflammation research. Also, it is necessary to take into consideration that keratinocytes and fibroblasts cultured separately behave differently than those in co-culture [36]. Moreover, in human skin, these cells communicate with each other via double paracrine signaling loops, which coordinate their actions to restore skin homeostasis after wounding [37].

**Figure 1:**
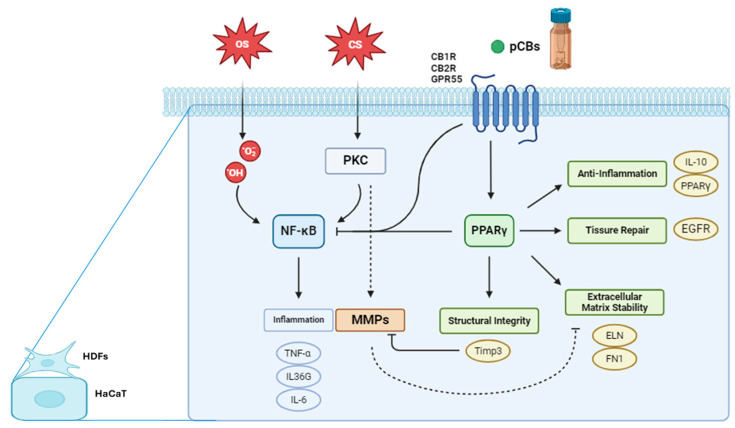
Schematic representation of gene expression pathways modulated by stressors and pCBs highlighting their potential effects on keratinocytes and fibroblasts. (1) Oxidative stress (OS) pathway. H_2_O_2_ generates an excess of ROS that triggers the activation of the NF-κB signaling cascade. NF-κB translocate from the cytoplasm to the cell nucleus, promoting the transcription of various pro-inflammatory genes, including TNF-α (a cytokine involved in systemic inflammation), IL-36G (a cytokine family involved in inflammatory responses), and IL-6 (with pro- and anti-inflammatory properties). (2) Chemical stress (CS) pathway. TPA activates PKC that (a) stimulates the NF-κB pathway, leading to the transcription of the same set of inflammatory genes as in the OS pathway, and (b) triggers a separate signaling cascade that results in the activation of MMPs that can significantly affect ECM stability, tissue integrity and cellular behavior. (3) Cannabinoid receptors, CB1R, CB2R, and GPR55. While pCBs cannabinoids may not always directly activate these receptors, they can influence their expression and modulate the ECS. The activation of the receptors leads to (a) stimulation of the PPARγ pathway, which can inhibit the inflammatory process by directly suppressing the NF-κB pathway, and (b) direct inhibition of the NF-κB pathway through multiple mechanisms, which may include preventing NF-κB translocation to the nucleus and interfering with NF-κB DNA binding. Solid arrows: direct activation or positive regulation; bar arrows: degradation or inhibitory steps; dashed arrows: secondary or indirect steps; blue boxes: components of the inflammatory process; brown box: MMPs process; blue circles: pro-inflammatory genes expressed; green boxes: components of the PPARγ pathway; yellow circles: anti-inflammatory and tissue repair genes expressed. Created using Biorender.com.

Figure 1 illustrates the interplay between oxidative stress, chemical stress, and the ECS in modulating gene expression pathways within keratinocytes and fibroblasts. Oxidative stress inducers, such as H_2_O_2_, lead to an overproduction of ROS that activate the NF-κB signaling pathway [38]. Once activated, NF-κB translocate to the cell nucleus, where it drives the transcription of pro-inflammatory genes, including TNF-α, IL-36G and IL-6 [39]. Chemical stressors, such as TPA, activate PKC, which in turn stimulates the NF-κB pathway, resulting in the transcription of the same inflammatory genes as seen in the oxidative stress pathway [40]. Additionally, PKC activation triggers another signaling cascade that leads to the activation of MMPs. These zinc-dependent enzymes degrade components of the ECM, such as collagens and elastin (ELN), which can compromise ECM stability, tissue integrity and cellular behavior [40]. Cannabinoid receptors, including cannabinoid receptor type 1 (CB1), cannabinoid receptor type 2 (CB2), and G protein-coupled receptor 55 (GPR55), play vital roles in regulating various skin physiological processes like inflammation, pain perception, cell proliferation and differentiation [41]. CB1 is linked with pain modulation, neuroprotection, and anti-inflammatory effects; CB2 is primarily involved in anti-inflammatory and immunomodulatory functions, and GPR55, sometimes referred to as the “third” cannabinoid receptor, influences inflammatory responses and cell proliferation. Although pCBs may not always directly activate these receptors, they can modulate receptor expression and thereby influence the ECS [30,41]. Activation of these receptors can lead to PPARγ pathway activation, which is crucial for anti-inflammatory responses, tissue repair and ECM maintenance. One significant gene regulated by PPARγ is TIMP3, which inhibits MMPs, preventing excessive ECM degradation and promoting tissue homeostasis. The activation of cannabinoid receptors and the PPARγ pathway can inhibit NF-κB by various mechanisms, such as preventing NF-κB from translocating to the nucleus, interfering with its DNA binding, or modulating upstream signals that activate NF-κB [30,42]. H_2_O_2_ induces oxidative stress, triggering cellular damage and responses such as inflammation, apoptosis, or senescence [43,44,45]. By its turn, TPA activates signaling pathways, particularly through PKC [40], influencing processes like cell proliferation, differentiation, and immune responses, often leading to inflammation and potential tumorigenesis [46,47,48]. Both stressors stimulate the production of pro-inflammatory cytokines, including IL-6, TNF-α, and IL-36G, which are key mediators in acute and skin-specific inflammatory responses. Counteracting these effects, anti-inflammatory markers like IL-10 and PPARγ play critical roles in reducing inflammation, limiting tissue damage and promoting tissue repair. Up-regulation of IL-10 and PPARγ is a promising therapeutic strategy for managing inflammation and enhancing healing.

## 2. Results and Discussion

### 2.1. Evaluation of Cellular Viability of Cell Lines Exposed to Stress-Inducing Agents: Optimization of the Experimental Conditions

In previously published work, the safety of pure pCBs, CBD, CBC, CBG, and pCBs-based formulations was evaluated following their incubation in HaCaT and HDF cell lines. The cell viability obtained was above 90% for all the pure pCBs and pCBs formulations. These results demonstrated the safety of pCBs formulations to be used in skin disorders associated with inflammation and cellular homeostasis [49,50].

For each inducing agent, different concentrations were tested, and the respective cell viability after incubation in the cell lines under study was evaluated. These preliminary assays allowed for the selection of the concentration of the inducing agent that would result in a loss of cell viability below 20%.

#### 2.1.1. H_2_O_2_

H_2_O_2_ is an oxidizing agent that induces cellular damage at inappropriate concentrations [51,52]. It has been extensively used in research to induce cellular oxidative stress aiming to modulate redox-regulated cellular pathways [53]. Its role as both a signaling molecule and a source of oxidative stress makes it crucial in understanding various skin conditions, their progression, and potential treatments. Fibroblasts, the cells responsible for producing collagen and other ECM components, react to H_2_O_2_ during wound healing. In keratinocytes, H_2_O_2_ can induce oxidative damage that disrupts the skin barrier function.

**Figure 2:**
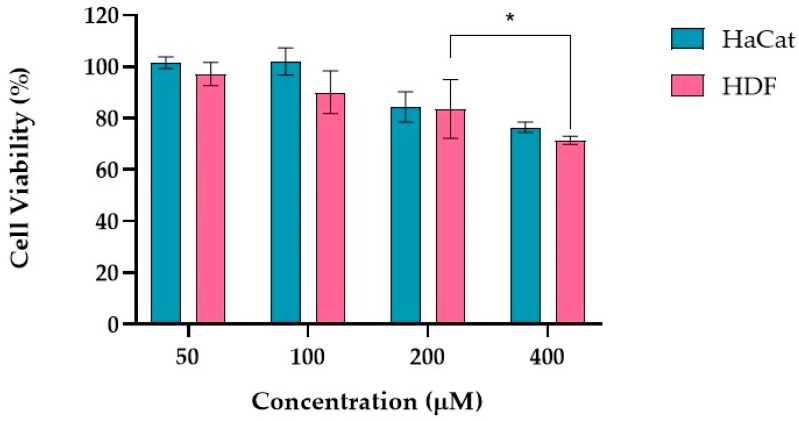
Assessment of cell viability of HaCaT and HDF exposed for 24 h to the stress-inducing agent, H_2_O_2_, tested over a concentration range between 50 and 400 µM. Results correspond to 6 replicates and 3 independent assays. * *p* < 0.05.

In Figure 2, the cell viability obtained for HaCaT and HDF cell lines incubated for 24 h with H_2_O_2_ is presented. The concentrations of H_2_O_2_ ranged from 50 to 400 μM. The cell viability at a concentration of 50 μM was above 97%, at 100 μM higher than 90%, at 200 μM around 84%, and at 400 μM higher than 71% for both skin cell lines tested.

For HDF cells, statistically significant differences were observed for the two higher concentrations: 200 and 400 μM. Based on these results, 200 μM was the H_2_O_2_ concentration chosen for the evaluation of the molecular variations after stress induction, as a reduction in cell viability below 20%, for the two cell lines under study, was achieved.

#### 2.1.2. TPA

TPA plays a critical role in various cellular pathways, including cell proliferation, differentiation, and apoptosis, and has the capacity to generate reactive oxygen species [54]. TPA is a potent activator of PKC, influencing multiple cellular processes, such as gene expression, modulation of the immune response, and cell cycle regulation. TPA is also recognized as a cellular stress factor, inducing oxidative stress by generating reactive oxygen species (ROS) and activating stress-related signaling pathways [55,56].

TPA is used to induce inflammatory responses in cell cultures and animal models, enabling the study of inflammatory pathways to evaluate anti-inflammatory therapies.

**Figure 3:**
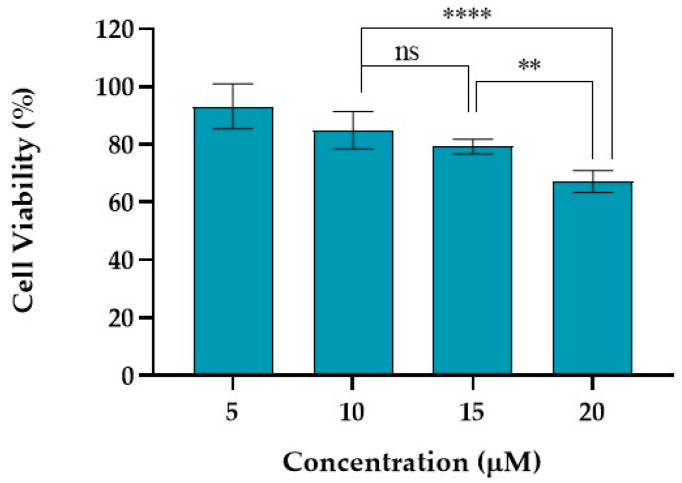
Assessment of cell viability of HaCaT exposed for 24 h to the stress-inducing agent, TPA, tested over a range of concentrations between 5 and 20 µM. Results correspond to 6 replicates and 3 independent assays. ** *p* < 0.005, **** *p* < 0.0001, ns—not statistically significantly different.

Figure 3 depicts the results obtained for the HaCaT cell line incubated for 24 h with TPA. The concentrations tested for TPA ranged from 5 to 20 μM. For a concentration of 5 μM, cell viability above 93% was achieved, while for 10 μM, the cell viability was superior to 84% for both cell lines. TPA at concentrations of 15 and 20 μM induced a HaCaT cell reduction of 21 and 33%, respectively.

Keratinocytes, due to their distinct physiological role and cellular characteristics, were the primary focus for the application of TPA in this study. They represent the epidermal layer, are the first line of defense, and are directly exposed to environmental and chemical stressors like TPA in vivo. This direct interaction provides critical insights into the initial inflammatory and stress responses. On the other hand, HDF cells reside in the dermis and are only indirectly exposed to such stressors, so their evaluation may be useful for studying adverse effects or for a broader tissue response. However, the primary focus on HaCaT cells is generally more relevant for assessing direct chemical stress effects.

Based on these results, the concentration selected for the evaluation of the molecular variations after stress induction for HaCaT was 10 μM, as a reduction in cell viability below 20% was achieved and no statistically significant differences were observed between 10 and 15 μM.

### 2.2. Evaluation of Gene Expression Markers in Cell Lines

#### 2.2.1. Molecular Insights into the Pro-Inflammatory Modulation by F1CR1, F1CR2, F2CAA, and F3TAC Formulations in Pre-Treatment and Treatment Conditions

**Figure 4:**
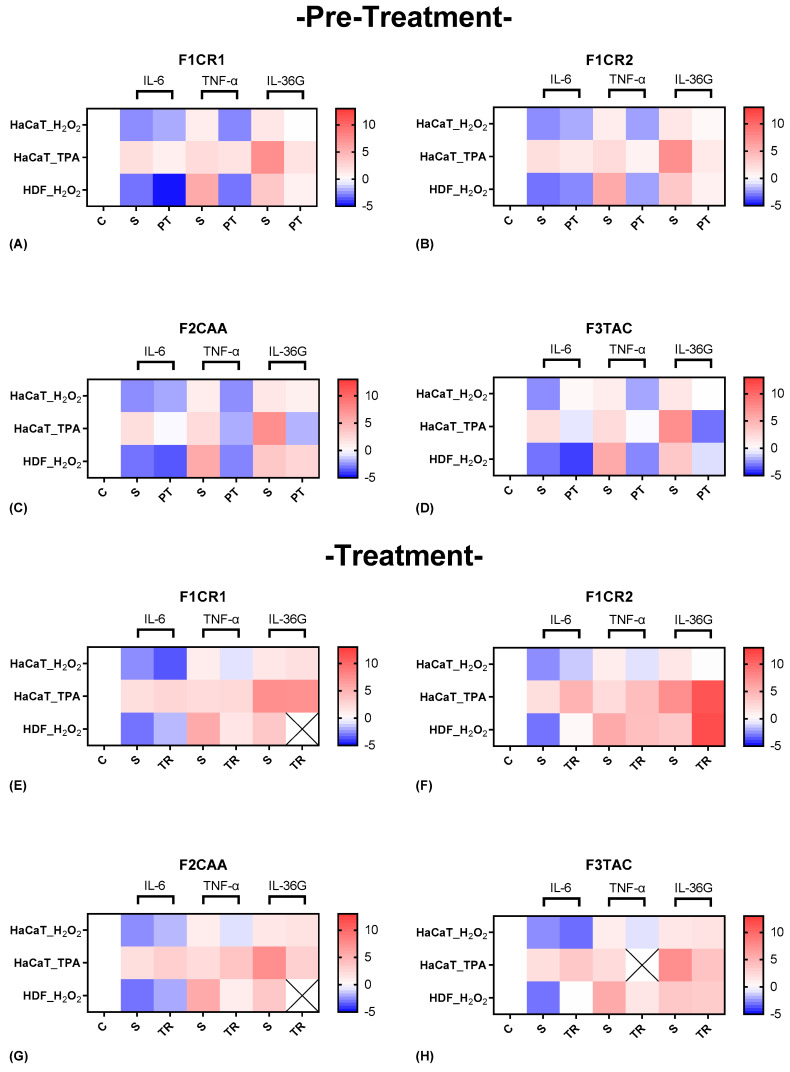
Heat map of pro-inflammatory genetic markers (IL-6, TNF-α and IL-36G) in HaCaT and HDF cells induced with H_2_O_2_ and TPA and pre-treated (**A**–**D**) and treated (**E**–**H**) with the formulations F1CR1, F1CR2, F2CAA and F3TAC. The blue color indicates downregulation of the gene, whereas the red represents upregulation. C = control, S = stress condition, PT = pre-treatment with formulation, TR = treatment with formulation, cross = no detectable expression level. Data are expressed as mean (log_2_ (fold change)) of sets of experiments for each condition (n ≥ 2).

The effect of each one of the proprietary formulations on pro-inflammatory markers (IL-6, TNF-α, and IL-36G) was assessed in HaCaT and HDF cells following exposition to H_2_O_2_ and TPA, either in pre-treatment or treatment conditions (Figure 4). Detailed results can be found in Supplementary Materials, “S1—Effect of formulations on pro-inflammatory markers”.

**F1CR1 Pre-Treatment:** IL-6, TNF-α, and IL-36G were significantly downregulated in both HaCaT and HDF cells across oxidative and inflammatory stress conditions, indicating strong anti-inflammatory potential and protection against skin damage (Figure 4A). These results suggest that F1CR1 effectively primes skin cells to tolerate oxidative and inflammatory stress, making it highly protective against skin damage.

**F1CR1 Treatment:** The key pro-inflammatory markers were reduced post-stress, highlighting its therapeutic role in managing ongoing skin inflammation and aiding recovery (Figure 4E). Overall, F1CR1 modulates both inflammatory and regenerative processes in HaCaT and HDF, offering protection and aiding recovery in various stress conditions.

**F1CR2 Pre-Treatment:** TNF-α and IL-36G were downregulated in HaCaT cells showing stress and cell-specific effects. TNF-α was strongly reduced in HDF cells, indicating modulation of inflammation and tissue repair (Figure 4B). This suggests that F1CR2 helps modulate the inflammatory response and offers protection against oxidative stress and highlights a tailored inflammatory response and prevents chronic skin damage under inflammatory conditions.

**F1CR2 Treatment**: The results varied depending on the type of stressor (Figure 4F). Post-oxidative stress reduced TNF-α in both cell types, promoting regeneration. However, post-TPA stress, upregulation of IL-6, TNF-α and IL-36G suggested reduced effectiveness in controlling advanced inflammation. This highlights the importance of timing when using F1CR2 for its anti-inflammatory and regenerative properties.

**F2CAA Pre-Treatment:** IL-6, TNF-α and IL-36G were downregulated in both HaCaT and HDF cells, showing strong anti-inflammatory effects and priming skin against inflammatory stress (Figure 4C).

**F2CAA Treatment:** Post-oxidative stress promotes a reduction in TNF-α and an upregulation of IL-6 in both cell types. After TPA-induced stress, IL-36G was downregulated, indicating a potential in targeting chronic inflammation pathways (Figure 4G). Overall, F2CAA demonstrates strong anti-inflammatory potential in both pre-treatment and treatment scenarios, with its ability to modulate key cytokines making it a valuable agent in managing skin inflammation.

**F3TAC Pre-Treatment:** IL-6, TNF-α and IL-36G were downregulated in HDF cells and showed significant anti-inflammatory effects under oxidative stress. Before TPA stress, all three markers were reduced in HaCaT cells, highlighting strong protective potential (Figure 4D).

**F3TAC Treatment**: TNF-α and IL-36G were downregulated in HaCaT and HDF cells, though with limited effectiveness compared to pre-treatment. IL-36G was strongly downregulated post-TPA stress, maintaining some effect in modulating inflammatory pathways (Figure 4H). These findings suggest that F3TAC has both protective and therapeutic properties depending on when it is administered.

#### 2.2.2. Molecular Insights into the Anti-Inflammatory Effects of F1CR1, F1CR2, F2CAA and F3TAC Formulations in Pre-Treatment and Treatment Conditions

**Figure 5:**
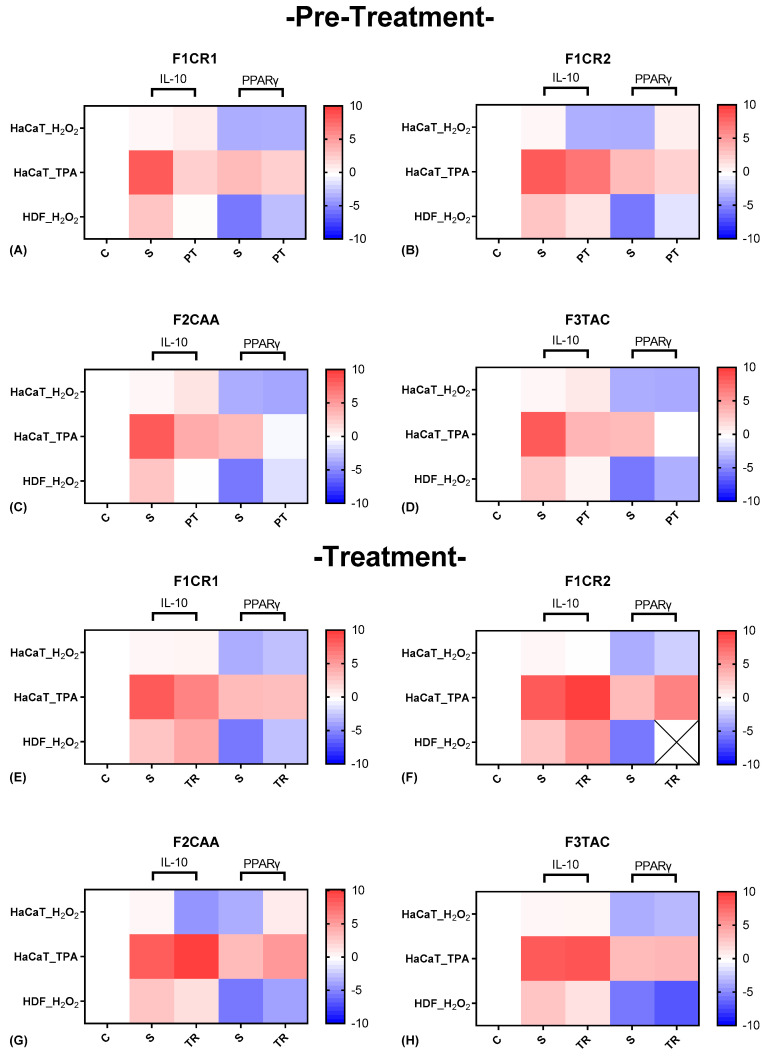
Heat map of anti-inflammatory genetic markers (IL-10 and PPARγ) in HaCaT and HDF cells induced with H_2_O_2_ and TPA and pre-treated (**A**–**D**) and treated (**E**–**H**) with the formulations F1CR1, F1CR2, F2CAA and F3TAC. The blue color indicates downregulation of the gene, whereas the red represents upregulation. C = control, S = stress condition, PT = pre-treatment with formulation, TR = treatment with formulation, cross = no detectable expression level. Data are expressed as mean (log_2_ (fold change)) of sets of experiments for each condition (n ≥ 2).

The effect of each one of the proprietary formulations on anti-inflammatory markers (IL-10 and PPARγ) was assessed in HaCaT and HDF cells following exposition to H_2_O_2_ and TPA, either in pre-treatment or treatment conditions (Figure 5). Detailed results can be found in Supplementary Materials, “S2—Effect of formulations on anti-inflammatory markers”.

**F1CR1 Pre-Treatment:** HaCaT cells exposed to H_2_O_2_ stress showed no significant changes in IL-10 or PPARγ, suggesting limited modulation of anti-inflammatory responses under oxidative stress (Figure 5A). In HDF cells, both IL-10 and PPARγ were significantly downregulated, indicating a shift in anti-inflammatory mechanisms in fibroblasts. Under TPA stress, IL-10 and PPARγ were significantly downregulated in both cell types, highlighting stress- and cell-specific effects.

**F1CR1 Treatment:** In HaCaT cells, H_2_O_2_ stress led to PPARγ downregulation, implicating modulation of inflammatory responses (Figure 5E). In HDF cells, IL-10 was upregulated, and PPARγ was strongly downregulated, indicating anti-inflammatory potential. Following TPA stress, HaCaT cells exhibited downregulation of IL-10, with no significant changes in PPARγ, reflecting a stress-specific compensatory mechanism.

**F1CR2 Pre-Treatment:** Both HaCaT and HDF cells showed strong downregulation of IL-10 and upregulation of PPARγ under H_2_O_2_ stress, highlighting enhanced stress tolerance and repair mechanisms (Figure 5B).

**F1CR2 Treatment:** In HaCaT cells, post-H_2_O_2_ stress resulted in no significant changes in IL-10 but maintained PPARγ upregulation, supporting recovery pathways (Figure 5F). In HDF cells, IL-10 was upregulated, with consistent PPARγ upregulation across cell lines, emphasizing anti-inflammatory and tissue repair capabilities.

**F2CAA Pre-Treatment:** In HaCaT cells exposed to H_2_O_2_ stress, IL-10 was slightly upregulated, suggesting enhanced anti-inflammatory responses (Figure 5C). In HDF cells, both IL-10 and PPARγ were downregulated, indicating a more targeted and cell-specific inflammation modulation. Under TPA stress, both IL-10 and PPARγ were downregulated in HaCaT cells, indicating stress-specific effects.

**F2CAA Treatment:** In HaCaT cells after H_2_O_2_ stress, there was a strong downregulation of both IL-10 and PPARγ, suggesting a more pronounced inflammatory modulation (Figure 5G). In HDF cells, IL-10 was downregulated, but PPARγ was upregulated, indicating an anti-inflammatory response. After TPA stress, HaCaT cells exhibited an upregulation of both IL-10 and PPARγ, indicating a protective response against inflammation. These results show that F2CAA’s effects are cell-type and stress-specific, with differing impacts on anti-inflammatory mediators.

**F3TAC Pre-Treatment:** In HaCaT cells, regarding H_2_O_2_ stress, there was a slight upregulation of IL-10 with no significant changes in PPARγ (Figure 5D). However, in HDF cells, both IL-10 and PPARγ were downregulated, indicating cell-specific effects of F3TAC in modulating pro- and anti-inflammatory signals in fibroblasts. Pre-treatment with F3TAC before TPA stress led to significant downregulation of both IL-10 and PPARγ in HaCaT cells, suggesting F3TAC’s effects are stress-dependent and may involve other compensatory mechanisms.

**F3TAC Treatment:** In HaCaT cells after H_2_O_2_ stress, it was denoted a slight but non-significant downregulation of IL-10 and PPARγ, suggesting limited effects after oxidative stress (Figure 5H). In HDF cells, there was a consistent downregulation of IL-10 and PPARγ, indicating a more pronounced effect on modulating anti-inflammatory pathways in fibroblasts. After TPA stress, HaCaT cells showed no significant changes in IL-10 or PPARγ, further indicating that F3TAC’s effects are context-dependent.

#### 2.2.3. Molecular Insights into the Structural Modulation by F1CR1, F1CR2, F2CAA and F3TAC Formulations in Pre-Treatment and Treatment Conditions

**Figure 6:**
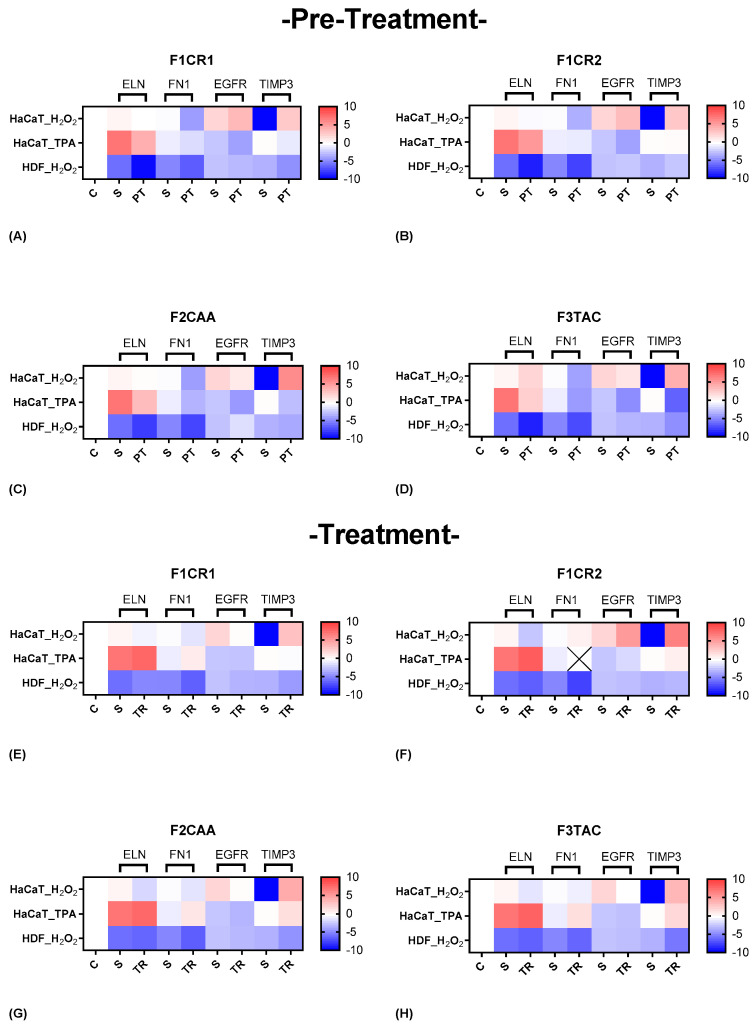
Heat map of structural genetic markers (ELN, FN1, EGFR and TIMP3) in HaCaT and HDF cells induced with H_2_O_2_ and TPA pre-treated (**A**–**D**) and treated (**E**–**H**) with the formulations F1CR1, F1CR2, F2CAA and F3TAC. The blue color indicates downregulation of the gene, whereas the red represents upregulation. C = control, S = stress condition, PT = pre-treatment with formulation, TR = treatment with formulation, cross = no detectable expression level. Data are expressed as mean (log_2_ (fold change)) of sets of experiments for each condition (n ≥ 2).

The effect of each one of the proprietary formulations on structural markers (ELN, FN1, EGFR and TIMP3) was assessed in HaCaT and HDF cells following exposition to H_2_O_2_ and TPA, either in pre-treatment or treatment conditions (Figure 6). Detailed results can be found in Supplementary Materials, “S3—Effect of formulations on structural markers”.

**F1CR1 Pre-Treatment:** In HaCaT cells exposed to H_2_O_2_ stress, FN1 was downregulated, while EGFR and TIMP3 were upregulated, promoting ECM modulation and cell survival (Figure 6A). In HDF cells, downregulation of ELN, FN1, and TIMP3 highlighted cell-specific responses aimed at preventing excessive collagen deposition associated with scarring or fibrosis. Reduced production of ELN and FN1 and decreased TIMP3 (which inhibits ECM degradation) collectively work to limit excessive ECM accumulation, a hallmark of fibrosis. Under TPA stress, HaCaT cells showed downregulation of ELN, FN1, TIMP3, and EGFR, reflecting protective effects against inflammation and excessive proliferation.

**F1CR1 Treatment:** After H_2_O_2_ stress, HaCaT cells exhibited FN1 downregulation and TIMP3 upregulation, potentially enhancing ECM remodeling (Figure 6E). HDF cells showed slight ELN upregulation, preserving elasticity, while FN1, EGFR, and TIMP3 were subtly downregulated. Post-TPA stress, HaCaT cells upregulated FN1, suggesting an enhanced wound-healing response.

**F1CR2 Pre-Treatment:** HaCaT cells under H_2_O_2_ stress downregulated ELN and FN1 and potentially prevented excessive ECM scaffolding, which could interfere with proper keratinocyte migration and re-epithelization, while EGFR and TIMP3 upregulation supported tissue repair (Figure 6B). In HDF cells, upregulation of ELN and FN1 indicated structural support and enhanced healing. F1CR2 pre-treatment primes HaCaT cells to resist oxidative stress and promote controlled wound repair. The differential effects observed in HDF cells indicate complementary roles between epidermal and dermal components, contributing to overall skin regeneration and minimizing the risk of abnormal scarring.

**F1CR2 Treatment:** After H_2_O_2_ stress, following H_2_O_2_ stress, HaCaT cells exhibited increased ECM production, as indicated by the upregulation of FN1, alongside enhanced tissue regeneration marked by elevated levels of EGFR and TIMP3 (Figure 6F). In contrast, HDF cells showed a significant downregulation of ELN and FN1, likely to mitigate the risk of excessive scarring, while a slight upregulation of TIMP3 suggested balanced matrix remodeling. After TPA-induced stress, HaCaT cells displayed an upregulation of ELN and TIMP3, with a modest downregulation of EGFR, reflecting an emphasis on structural support and ECM stabilization in response to inflammatory stress.

**F2CAA Pre-Treatment:** HaCaT cells under H_2_O_2_ stress strongly downregulated FN1 and EGFR, while TIMP3 was strongly upregulated, protecting against oxidative stress (Figure 6C) probably by reducing ECM degradation and potential excessive fibrosis or scarring. In HDF cells, downregulation of ELN and FN1 with EGFR upregulation highlighted fibroblast-specific responses.

**F2CAA Treatment:** HaCaT cells post-H_2_O_2_ stress downregulated ELN, FN1, and EGFR, with strong TIMP3 upregulation potentially protecting the ECM (Figure 6G). HDF cells showed downregulation of FN1, ELN, and TIMP3, reflecting complex ECM modulation. Post-TPA stress, HaCaT cells upregulated ELN, FN1, and TIMP3, enhancing tissue repair while moderating cell proliferation.

**F3TAC Pre-Treatment:** HaCaT cells under H_2_O_2_ stress upregulated ELN, enhancing elasticity, while FN1 and EGFR were strongly downregulated, reducing proliferation (Figure 6D). TIMP3 was strongly upregulated, providing robust ECM protection. In HDF cells, ELN, FN1, and TIMP3 were downregulated, indicating reduced ECM remodeling, with stable EGFR maintaining cell proliferation.

**F3TAC Treatment:** Post-H_2_O_2_ stress, the EGFR downregulation suggested reduced proliferation, and TIMP3 upregulation highlighted enhanced matrix protection in HaCaT cells (Figure 6H). In HDF cells, the downregulation of ELN, FN1, and TIMP3 indicated reduced matrix production and remodeling, with stable EGFR maintaining proliferation. After TPA stress, F3TAC-treated HaCaT cells showed upregulation of ELN, TIMP3, and slight FN1, suggesting enhanced ECM protection and production, while EGFR stability reflected consistent proliferative capacity.

#### 2.2.4. Molecular Evaluation of Cannabinoid Receptor Modulation by F1CR1, F1CR2, F2CAA and F3TAC Formulations in Pre-Treatment and Treatment Scenarios

**Figure 7:**
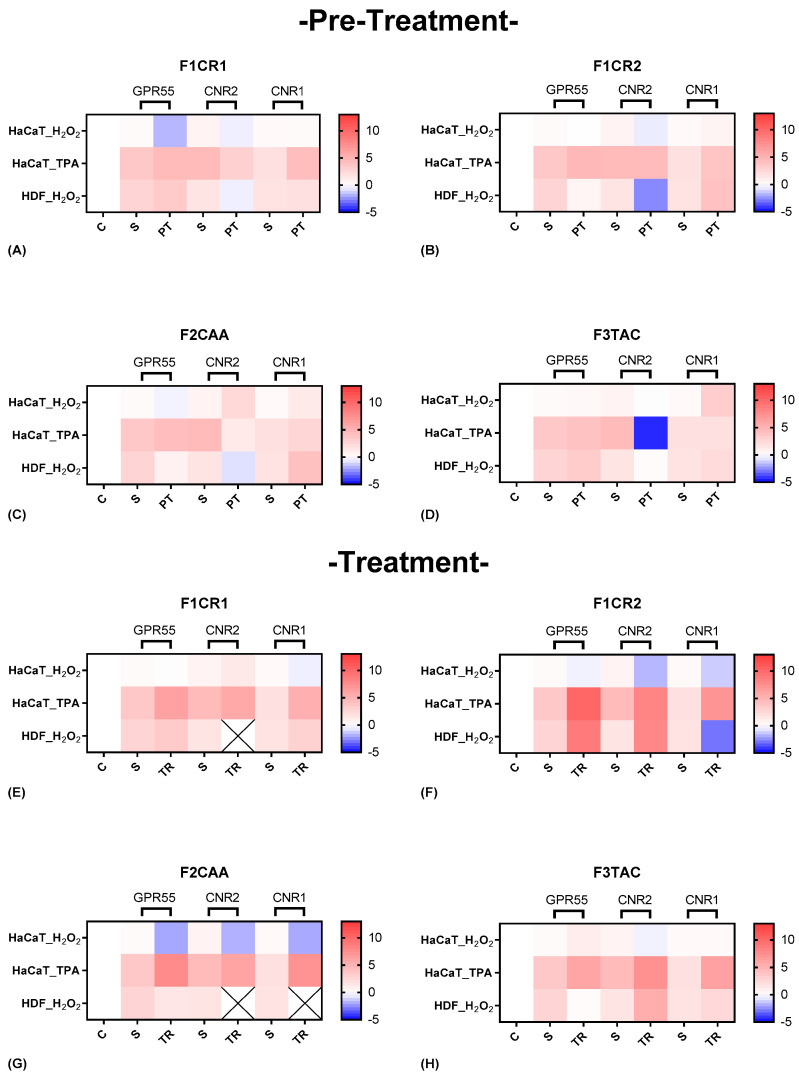
Heat map of cannabinoid receptors genetic markers (GPR55, CNR2 and CNR1) in HaCaT and HDF cells induced with H_2_O_2_ and TPA and pre-treated (**A**–**D**) and treated (**E**–**H**) with the formulations F1CR1, F1CR2, F2CAA and F3TAC. The blue color indicates downregulation of the gene, whereas the red represents upregulation. C = control, S = stress condition, PT = pre-treatment with formulation, TR = treatment with formulation, cross = no detectable expression level. Data are expressed as mean (log_2_ (fold change)) of sets of experiments for each condition (n ≥ 2).

The effect of each one of the proprietary formulations on cannabinoid receptor markers (GPR55, a gene that encodes CB2 (CNR2), and a gene that encodes CB1 (CNR1)) was assessed in HaCaT and HDF cells following exposition to H_2_O_2_ and TPA, either in pre-treatment or treatment conditions (Figure 7). Detailed results can be found in Supplementary Materials, “S4—Effect of the formulations on cannabinoid receptor markers”.

**F1CR1 Pre-Treatment:** Exposed to H_2_O_2_ stress, GPR55 and CNR2 were downregulated in HaCaT cells, suggesting potential anti-inflammatory actions, while CNR1 remained unchanged (Figure 7A). In contrast, HDF cells showed an upregulation of GPR55 and downregulation of CNR2, highlighting cell-specific responses, with GPR55 potentially aiding wound healing in fibroblasts. Under TPA-induced stress, HaCaT cells experienced upregulation of GPR55 and downregulation of CNR2, reinforcing the stress-specific nature of F1CR1’s effects.

**F1CR1-Treatment:** After H_2_O_2_ treatment, F1CR1 upregulated CNR2 and downregulated CNR1 in HaCaT cells, indicating modulation of HaCaT inflammation, while HDF showed mild upregulation of GPR55 and CNR1 (Figure 7E). Post-TPA treatment, all three receptors—GPR55, CNR2, and CNR1—were upregulated, suggesting F1CR1 activates the ECS to reduce inflammation and support regeneration under inflammatory stress. These results highlight the complex and context-dependent effects of F1CR1 on the ECS in skin cells.

**F1CR2 Pre-Treatment:** The application of F1CR2 prior to H_2_O_2_-induced oxidative stress in HaCaT cells resulted in the downregulation of GPR55 and CNR2, accompanied by an upregulation of CNR1 (Figure 7B). The downregulation of GPR55 suggests that F1CR2 may be attenuating pro-inflammatory effects, while the upregulation of CNR1 points to enhanced protective mechanisms against oxidative stress. In HDF cells, F1CR2 led to a strong upregulation of GPR55 and CNR2, with downregulation of CNR1, indicating activation of pathways involved in wound healing and tissue remodeling.

**F1CR2-Treatment:** In HaCaT cells, after H_2_O_2_-induced stress, CNR1 remained upregulated, but CNR2 and GPR55 were downregulated, indicating a reduced pro-inflammatory response (Figure 7F). In HDF cells, GPR55 and CNR2 were upregulated, promoting regenerative and anti-inflammatory responses, while CNR1 was downregulated, potentially indicating a shift towards cellular repair. This highlights that the timing of F1CR2 application is crucial for maximizing its anti-inflammatory and regenerative benefits.

**F2CAA Pre-Treatment:** In HaCaT cells, and before H_2_O_2_ stress, GPR55 was downregulated while CNR1 and CNR2 were upregulated, indicating a protective, anti-inflammatory effect (Figure 7C). In HDF cells, F2CAA pre-treatment also downregulated GPR55 and CNR2, with CNR1 upregulated, suggesting cell-specific protective responses. Under TPA stress, F2CAA caused GPR55 upregulation and CNR2 downregulation in HaCaT cells, pointing to a modulation of inflammatory pathways depending on the stress type.

**F2CAA Treatment:** Treatment with F2CAA, after H_2_O_2_ stress, led to the downregulation of all three receptors in HaCaT cells, suggesting a focus on reducing oxidative stress effects (Figure 7G). In HDF cells, only GPR55 was slightly downregulated, indicating a milder modulation. After TPA stress, F2CAA caused upregulation of all three receptors, showing enhanced cannabinoid signaling and tissue repair post-inflammation. These results emphasize the importance of timing when administering F2CAA, as it appears to provide both protective and regenerative benefits depending on when it is applied.

**F3TAC Pre-Treatment:** In HaCaT cells, and before H_2_O_2_ stress, GPR55 and CNR2 were slightly upregulated, and CNR1 was strongly upregulated, suggesting protection against oxidative stress through anti-inflammatory and antioxidant effects (Figure 7D). In HDF cells, F3TAC pre-treatment led to slight upregulation of GPR55 and CNR1, with downregulation of CNR2, indicating cell-specific protective mechanisms in the dermal layer. Under TPA stress, HaCaT cells pre-treated with F3TAC showed upregulation of GPR55, downregulation of CNR2, and unchanged CNR1, reflecting modulation of stress-specific inflammatory pathways.

**F3TAC Treatment:** In HaCaT cells, and after H_2_O_2_ stress, CNR2 was significantly downregulated, GPR55 was slightly upregulated, and CNR1 remained unchanged, indicating a shift in inflammatory modulation and regeneration post-stress (Figure 7H). In HDF cells, GPR55 was downregulated, while CNR1 and CNR2 were strongly upregulated, enhancing cannabinoid signaling and promoting anti-inflammatory and regenerative responses. Post-TPA treatment in HaCaT cells led to significant upregulation of all three receptors, suggesting enhanced cannabinoid signaling for repair and recovery from inflammatory stress.

The most noticeable results are indicated in Table 1, highlighting the potential pathways involved in the modulation of pro-inflammatory, anti-inflammatory, and cannabinoid receptor markers and ECM regulation suggested from the in vitro assays.

The observed effects across different cell lines suggest that F1CR1, F1CR2, F2CAA and F3TAC formulations may influence cell-specific and stress-dependent responses, highlighting their capacity to modulate inflammatory and regenerative processes in a controlled environment. These findings point to possible mechanisms that could be further explored for their therapeutic relevance.

### 2.3. In Vivo Efficacy Assays in Human Volunteers: A Proof of Concept

The regenerator cream, formulated with the **F1CR1** formulation, demonstrated significant efficacy in promoting skin recovery and regeneration across multiple dimensions. This aligns with the previously discussed properties of F1CR1, which include potent anti-inflammatory effects, enhancement of cellular repair mechanisms, and modulation of key structural and inflammatory pathways.

It was possible to demonstrate a statistically significant reduction in erythema recovery time, decreasing it by 2.7 days (12%) compared to the control (*p* = 0.034). This outcome highlights its efficacy in accelerating skin redness reduction after Sodium Lauryl Sulfate (SLS) exposure. This aligns with its intended use for enhancing regeneration in irritated skin. This outcome is consistent with F1CR1’s ability to downregulate pro-inflammatory cytokines, such as TNF-α, which are responsible for prolonged inflammation and redness in the skin.

Also, there was a significant improvement in microcirculation recovery time, reducing it by 1.9 days (6.6%) compared to the control (*p* = 0.033). The faster normalization of blood perfusion indicates reduced local inflammation and enhanced tissue repair. The cream enhanced the skin’s barrier function, significantly shortening the time by 2.7 days (11%) relative to the control (*p* = 0.031). This result underscores the cream’s ability to restore skin barrier function effectively, crucial for maintaining hydration and protecting against environmental stressors.

Participants in the self-assessment reported significant improvements in overall skin quality following the use of the product. A majority of 93% noted a visible reduction in redness, while 78.6% observed enhanced skin appearance, hydration and softness. Additionally, 64.3% of participants reported that their skin felt repaired after application. Notably, the product’s texture, absorption, and ease of application received unanimous praise, with 100% of users expressing satisfaction with these attributes. These subjective findings align with the instrumental results, reinforcing the product’s efficacy and user acceptability.

Regarding the **FC1R2** cream, while it was demonstrated that there was a reduced erythema recovery time by 2.2 days (9.5%), the result was not statistically significant (*p* = 0.140). Although less effective than FC1R2, it still showed potential for mitigating redness after SLS irritation.

LDF measurements indicated a non-significant decrease in microcirculation recovery time by 0.8 days (1.6%) compared to the control (*p* = 0.329). This suggests a lesser impact on reducing local inflammation and restoring normal blood flow than F1CR1.

TEWL recovery time was reduced by 1.4 days (5.4%), though the result was not statistically significant (*p* = 0.149), exhibiting a modest ability to improve skin barrier repair compared to F1CR1 and the control.

Participants in the self-assessment highlighted significant improvements in their skin condition after using the product. A majority of 93% reported a noticeable reduction in redness, while 86% experienced enhanced hydration and skin repair. Additionally, 86% noted that their skin felt softer and calmer following application. The product’s texture and ease of application received universal acclaim, with 100% of participants expressing appreciation for these attributes. These findings underscore the product’s effectiveness and high level of user satisfaction.

Both F1CR1 and F1CR2 creams demonstrated excellent skin compatibility. Neither cream caused adverse reactions or signs of irritation throughout the study period. Participants consistently rated the tolerability of both products as “very good”, reinforcing their suitability for sensitive or compromised skin.

The application of the **F2CAA** cream yielded measurable improvements in various skin parameters, demonstrating its efficacy in promoting skin health and addressing visible signs of aging.

The cream led to a modest yet consistent reduction across several spot metrics: a 3.6% decrease in visible spot count, a 5.6% reduction in visible spot area, a 4.3% reduction in UV spot area, and a 3.7% decrease in red spot count. Antioxidant capacity was evaluated using β-carotene as a marker, where the untreated control area exhibited a 53.6% greater color variation compared to the treated area following UVA-induced oxidation. This statistically significant reduction (*p* < 0.05) highlights the cream’s ability to mitigate oxidative stress and protect against UVA-induced damage.

The cream significantly reduced wrinkles and fine lines by 21.2% (*p* = 0.001). Clinical evaluation using the crow’s feet score further confirmed this finding, with a 9.5% reduction (*p* < 0.05) observed after 28 days. In addition, skin roughness decreased significantly, and skin firmness improved by 11.9% (*p* = 0.004). While skin elasticity showed a slight increase of 1.2%, this change was not statistically significant.

The cream demonstrated a 21.4% reduction in maximum microcirculation values and a 40.6% increase in vasodilation onset time following histamine iontophoresis. These statistically significant results (*p* < 0.05) indicate the cream’s capacity to delay and reduce vasodilation, underscoring its soothing properties. The delayed vasodilation reflects reduced inflammation and enhanced skin resilience to irritants.

The study reported a significant 19.1% decrease in TEWL (*p* = 0.003), indicating an improved skin barrier. Enhanced barrier function is critical for reducing inflammatory responses and protecting against environmental aggressors. Moreover, hydration levels increased significantly by 32.3% (*p* = 0.002), further supporting the cream’s efficacy in improving skin moisture retention and barrier integrity.

Collectively, these statistically significant findings validate the cream’s effectiveness in improving skin texture, hydration, and barrier function while providing protection against oxidative stress and inflammation. Statistical rigor ensured reliability and reproducibility, establishing robust evidence for the product’s performance.

Instrumental results were supported by subjective evaluations, with high participant satisfaction in hydration, texture, firmness and overall skin quality. Specifically, 100% of participants reported that their skin felt softer, 86% noted immediate hydration, 93% observed that their skin appeared hydrated, glowing, and radiant, 86% felt their skin was firmer, 64% noted a reduction in the appearance of lines and wrinkles, 86% reported that their skin felt restored and rejuvenated, 71% agreed that their skin looked and felt plumped and youthful, 86% felt their skin was nourished, and 93% agreed that their skin felt energized, brighter, and that its texture was improved and more even. Additionally, 50% observed a reduction in hyperpigmentation, 86% noticed a reduction in pore size, and 64% reported a reduction in skin redness. Regarding the cosmetic formulation, 100% of participants expressed satisfaction with the texture and found the product easy to absorb and spread.

Product compatibility was rated “Very Good”, with no adverse reactions reported. The study validates the efficacy of the tested product in improving skin health metrics, offering significant benefits in hydration, elasticity, barrier integrity and antioxidative protection.

Regarding the application of **F3TAC**, the study results provided a comprehensive analysis of various skin parameters, reflecting subtle changes in response to the treatment. The skin lipidic index exhibited a modest 9.6% increase, though this change was not statistically significant (*p* > 0.05), suggesting minor adjustments in sebum levels. Skin hydration improved by 12.9%, but this too lacked statistical significance (*p* > 0.05), indicating potential hydration benefits that warrant further exploration.

TEWL values showed a 20.7% increase, which, while not statistically significant (*p* > 0.05), may reflect early adaptations in the skin barrier function, emphasizing the need for additional investigation into long-term effects. Regarding red spots, an 8.7% increase in count and a 5.1% increase in area were noted, though these changes did not reach statistical significance. Visible spots demonstrated a 1.6% decrease in count and a 6.4% increase in area, reflecting minor and statistically non-significant variations (*p* > 0.05).

For skin pores, a 3.0% decrease in count and a 5.8% reduction in area were observed, though neither result was statistically significant. Finally, porphyrins, indicative of bacterial by-products, showed a notable 10.4% reduction in both count and area, albeit without statistical significance (*p* > 0.05), suggesting a potential improvement in the skin’s control of microbial activity. These findings collectively highlight subtle, early-stage changes in skin metrics, providing a foundation for further research into the treatment’s efficacy.

A possible explanation for these outcomes can be that sometimes, topical treatments, especially those targeting acne, can trigger an initial purging phase. This is a period where the skin begins to expel impurities from beneath the surface, leading to a temporary increase in spots and redness. The increase in red spots and their area could be indicative of this purging process, where the skin is reacting to the active ingredients in the tonic by bringing underlying issues to the surface. Moreover, a positive outcome was that there was a 10.4% decrease in both porphyrin count and area. Porphyrins are produced by bacteria such as *Cutibacterium acnes* and are known as virulence factors involved in the pathogenesis of acne [57,58]. High levels are associated with increased acne severity, whereas a reduction in porphyrins correlates with acne improvement [59].

While the instrumental results indicated subtle changes in skin parameters, such as a 9.6% increase in the skin lipidic index and a 12.9% improvement in hydration (both not statistically significant), participants reported positive subjective experiences. Notably, 67% of participants felt their skin appeared brighter, felt protected, and experienced reduced irritation with regulated sebum secretion, complementing the observed modest increase in lipidic index.

Similarly, while the TEWL increase of 20.7% suggested potential early adjustments in the skin barrier, 58% of participants noted their skin felt more balanced, with improvements observed as early as the fifth day.

The 10.4% reduction in porphyrins, indicative of controlled bacterial by-products, correlated with 75% of participants reporting reduced blemishes and breakouts and 67% noting faster disappearance of blemishes. These findings reinforce the tonic’s role in managing skin microbiota and reducing irritation.

Although the changes in visible and red spot metrics did not reach statistical significance, participants’ subjective feedback was encouraging. For instance, 58% reported reduced scarring associated with blemishes or acne, and 50% agreed that pore size and redness were reduced, aligning with the 3.0% decrease in pore count and 5.8% reduction in pore area observed instrumentally.

Furthermore, 41% of participants noticed a reduction in hyperpigmentation, despite only modest variations in visible spot metrics.

The cosmetic formulation’s appeal was also evident, with 92% of participants expressing satisfaction with the texture and 83% noting that the product absorbed easily.

These findings underscore the tonic’s potential as a cosmetically elegant and user-friendly formulation that delivers perceptible benefits, complementing the instrumentally measured improvements in skin health. Together, the subjective and objective data highlight the tonic’s multifaceted efficacy in addressing skin concerns and enhancing overall user satisfaction.

The study demonstrated that the tonic was highly compatible with participants’ skin, as no adverse reactions were reported. Compatibility was rated as “Very Good” by all participants, underscoring the product’s safety profile.

In conclusion, while the product produced changes across all measured parameters, these differences were not statistically significant; the results indicate a trend towards a reduction in pore appearance and porphyrins, aligning with the intended purpose of an anti-acne tonic. To enhance the efficacy of the tonic, several strategies could be considered, focusing on both formulation improvements and complementary approaches; by increasing the concentration of key active ingredients (within safe limits), it can be combined with F1CR1 and/or tested for more time. The efficacy of acne treatments should typically be monitored over a period of 8 to 12 weeks, as the skin cycle, or the time it takes for new skin cells to form and reach the surface, is about 4 to 6 weeks. Monitoring for at least two full skin cycles allows for a more accurate assessment of how well the treatment is working. Most topical acne treatments require several weeks to start showing noticeable effects. Initial improvements in acne lesions, reduction in inflammation, and overall skin texture often become evident after 4 to 8 weeks of consistent use.

## 3. Materials and Methods

### 3.1. Materials

Phosphate-buffered saline (PBS), 3-(4,5-Dimethylthiazol-2-yl)-2,5-diphenyltetrazolium bromide (MTT) and hydrogen peroxidase (H_2_O_2_) were obtained from Sigma-Aldrich (St. Louis, MO, USA). TRK Lysis Buffer was acquired from VWR Life Sciences (Radnor, PA, USA). The culture media, fetal bovine serum (FBS), antibiotics and phorbol 12-myrstate 13-acetate (TPA) were purchased from Invitrogen (Thermo Fisher Scientific, Waltham, MA, USA). All other chemicals and reagents were of analytical grade.

### 3.2. Cell Line Culture Conditions

Human keratinocytes (HaCaT) (CLS 300493) and human dermal fibroblasts (HDF) (ATCC^®^ PCS-201-012™) were maintained in Dulbecco’s Modified Eagle’s Medium (DMEM) with high glucose (4500 mg/L), supplemented with 10% FBS and 100 IU/mL of penicillin and 100 μg/mL of streptomycin, henceforth designated as complete culture medium. All cells were maintained at 37 °C in a 5% CO_2_ environment, the mediums were changed, and the cells were trypsinized every 3–4 days when 80% confluence was reached.

### 3.3. Evaluation of Cellular Viability of Cell Lines Exposed to Stress-Inducing Agents

The MTT assay was used to evaluate the cell viability of HaCaT and HDF in the presence of increasing concentrations of two stress inducers, namely H_2_O_2_ and TPA. Briefly, cells were seeded at a concentration of 2 × 10^4^ cells/mL (200 μL) in 96-well plates and allowed to adhere for 24 h. On the next day, the medium was removed, and cells were incubated with stress inducers for 24 h at 37 °C in a humidified air incubator under a 5% CO_2_ atmosphere. After that, the culture medium was removed, and the cells were washed twice with PBS. Then, 50 μL of an MTT solution at 0.5 mg/mL in incomplete medium was added to the cells and allowed to incubate for 2–4 h. Later, 200 μL of DMSO was added in order to solubilize the formazan crystals produced by the cells upon MTT reduction. The absorbance was then read at 570 nm using a BioTek ELx800 Absorbance Microplate Reader (BioTek Instruments, Inc., Winooski, VT, USA), and the cells’ viability (%) was calculated according to the following equation:

(1) $$Cell\;viability\left(\%\right)=\frac{{OD}_{t}}{{OD}_{c}}\times 100$$

where OD_t_ is the optical density of the cells from testing groups and OD_c_ is the optical density of control cells (cells in complete medium corresponding to 100% cell viability).

Table 2 describes the range of concentrations for the stress inducers tested for each cell line.

### 3.4. Formulation Development and Analysis

pCBs isolated from *Cannabis sativa* provided the source for 4 proprietary formulations: F1CR1, F1CR2, F2CAA and F3TAC (Table 3). CBD was obtained from BSPG (https://www.bspglab.co.uk/, accessed on 4 January 2023), CBG and CBC from ECS Brands (https://ecsbrands.com/isolates/, accessed on 4 January 2023). Also, purified CBG and CBC samples (>99.5%) were obtained by centrifuge partition chromatography (CPC) technology using an rCPC device (RotaChrom, Purified Solutions, Budapest, Hungary). The method was internally developed through optimizing the best solvent combination alongside the most appropriate solvent ratio (proprietary data). Briefly, CPC is a liquid–liquid preparative chromatographic technique that makes use of two immiscible liquid phases, the solvent system, representing the stationary and mobile phases of a typical chromatographic apparatus. The main goal of this technology is to isolate specific compounds with a high purity grade (>95%) and high recovery mass yield (>85%). Formulations were kept in dark amber glass flasks at room temperature. The cannabinoids and formulations were analyzed by high-performance liquid chromatography (HPLC, Cannabis Analyzer™ for Potency, Shimadzu, Kyoto, Japan).

The 4 proprietary formulations were designed with the potential for anti-inflammatory and regenerative actions (F1CR1, F1CR2 and F2CAA) and additionally for anti-acne (F3TAC). The formulations contain different ratios between each one of the cannabinoids. The ratio and concentration of cannabinoids in the formulations are proprietary information and cannot be disclosed to protect intellectual property and competitive advantage.

The cosmetic matrices used in this study were expertly produced by a specialized cosmetic company (COSMETEK-Cosmetic Lab & Consulting, https://www.cosmetek.org/sobre-nos/, accessed on 1 February 2023) ensuring high-quality and standardized matrices.

### 3.5. Evaluation of pCBs Effect in Pre-Treatment and Treatment Conditions

The effect of pCBs-based formulations on gene expression in the studied cell lines, HaCaT and HDF, was evaluated under both prophylactic (pre-treatment) and therapeutic (treatment) conditions. In these two approaches, stress was induced either after (pre-treatment) or before (treatment) incubation with the pCBs formulations. The molecular response under all experimental stress conditions was assessed by measuring the expression of relevant genes using qRT-PCR.

For the gene expression assay, cells were seeded at a concentration of 1.7 × 10^5^ cells/mL (3 mL) in 6-well plates and allowed to adhere for 24 h.

- Pre-treatment effect

**Figure 8:**
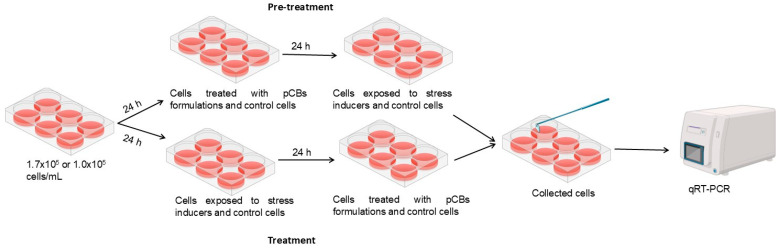
Scheme of the two in vitro schedules (pre-treatment and treatment) used to assess the ability of pCBs formulations to modify gene expression to basal levels.

Cells were allowed to adhere for 24 h. After this period, the supernatant was removed, and pCBs formulations solubilized in complete medium were added [49,50]. Complete medium was also added to negative and positive controls. After 24 h, the medium was discarded in all wells, and stress inducer solution was added in tested wells and positive control and complete medium in negative control. In Figure 8, a scheme of the protocol is represented.

- Treatment effect

Cells were allowed to adhere for 24 h. After this period, the medium was discarded, and cells were incubated with stress inducer at the pre-selected concentrations or complete medium (negative control). After a 24 h incubation period, the medium was discarded, and the cells were incubated with pCBs formulations [49,50] or with complete medium, either for negative (control cells) or positive controls (cells incubated with stress inducers).

After 24 h the supernatant was removed, and cells were washed with PBS twice to remove cellular debris. Cells were removed from each well, collected in pairs and centrifuged at 1000× *g*, for 20 min. at 4 °C. The pellet was then lysed with TRK lysin buffer, and 2-Mercaptoethanol was added to inhibit RNase activity.

The concentrations of the stress-inducing agents were chosen based on previous cell viability results performed in 96-well plates. Therefore, the concentration of H_2_O_2_ used in HaCaT and HDF cells was 1700 μM. The stress inducer TPA was tested in the HaCaT cell line at a concentration of 85 μM.

### 3.6. Real-Time Polymerase Chain Reaction (RT-qPCR)

**Figure 9:**
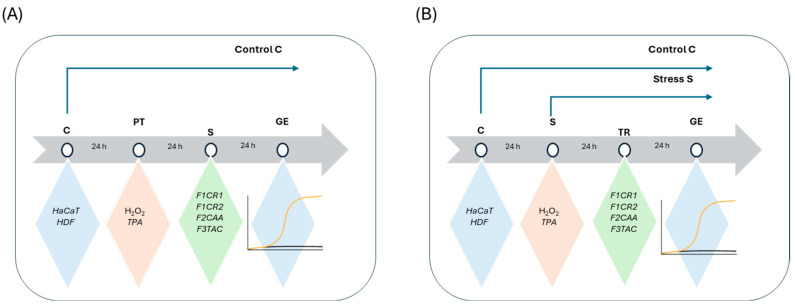
Scheme illustrating the time points used in the in vitro assays for (**A**) pre-treatment and (**B**) treatment, designed to evaluate the efficacy of pCBs formulations and their impact on gene expression modulation. Created using Biorender.com.

The gene expression level for each of the genes selected as a molecular marker was determined by real-time quantitative PCR using TaqMan probes (Thermo Scientific, Waltham, MA, USA) (Figure 9). The quantitative real-time PCR reaction allowed the quantification of the gene expression of the genes of interest. This method combines PCR amplification technology with real-time product detection and quantification using fluorophores. The advantage of this technique is that it relies on the amount of product formed in the exponential phase, where the DNA amplification reaction conditions are optimal with a strong correlation between the threshold and the initial amount of DNA in the test sample.

Total RNA was extracted from different cell lines and exposed to several compounds and pCBs-based formulations using a commercial kit according to the manufacturer’s instructions (Thermo Scientific), and after extraction, the mRNA was quantified with a NanoDrop. cDNA synthesis was performed using a commercial kit that includes random primers (Thermo Scientific); the isolated mRNA was reverse transcribed in a final volume of 20 µL, following incubation in a thermocycler for 5 min at 22 °C, 30 min at 42 °C and 5 min at 85 °C.

Twelve sets of primers and probes were designed based on the human genome (ENSEMBL Genome Browser) using Primer Express Software (3.0.1, Thermo Fisher Scientific, USA). The used primers are identified in Table 4.

RT-qPCR was performed using the PerfeCTa qPCR FastMix II Low ROX in the QuantStudio™ 5 Real-Time PCR System. Each PCR reaction (20 µL final volume) was carried out with 5 µL of cDNA, 0.4 µL of primers Forward + Reverse (200 nM mix), and 0.4 µL probe (200 nM). The qPCR reaction was performed with the following conditions: holding at 95 °C for 5 min, cycling at 95 °C for 30 s and 54 °C for 1 min (for 45 cycles). The relative expression of the different amplicons was calculated using the delta–delta Ct (∆∆Ct) method and then converted into the relative expression ratio (2^−∆∆Ct^) and log_2_(2^−∆∆Ct^) for graphic elaboration. All data were normalized to the endogenous reference gene rRNA18S.

### 3.7. In Vivo Efficacy and Safety Studies

The efficacy of the formulations F1CR2, F1CR1, F2CAA and F3TAC was assessed through the development of two regenerators, one anti-aging cream, and an anti-acne tonic. Each product was infused with pCBS as the primary active ingredient (Section 3.4). To enhance their effectiveness, additional active ingredients were selected based on their specific benefits. For acne treatment, salicylic acid was incorporated at low concentrations for its well-known acne-control properties. Essential oils, including lavender, juniper, and tea tree oil, were added selectively to the creams for their antimicrobial and soothing effects.

A single-center, blinded, randomized and controlled study was conducted to evaluate the efficacy, compatibility, and acceptability of the creams and tonic. This study, carried out by PhdTrials (https://phdtrials.com/, accessed on 12 March 2024) in accordance with Good Clinical Practices and NP EN ISO 9001:2015 [60] certification, also evaluated the cosmetic qualities and subjective efficacy of the three products. Ethical considerations aligned with the Declaration of Helsinki and relevant local legal frameworks were rigorously observed throughout the study. All assessments were carried out under controlled environmental conditions, maintaining a consistent temperature of 21 °C ± 2 °C and relative humidity of 55% ± 10%. Participants received standardized training on product application, with usage monitored to ensure accuracy and consistency. The study protocol was closely followed, with regular monitoring to identify and address any deviations promptly, ensuring the integrity and reliability of the results.

Regarding the F1CR1 and F1CR2 formulations and the associated creams, the study aimed to evaluate the regenerative efficacy of the two cosmetic formulations under controlled conditions. The study involved 14 participants who met specific inclusion criteria: aged 18–35 years, both genders, and free from contraindications such as recent topical treatments, allergies, or excessive sun exposure. The skin irritation model involved Sodium Lauryl Sulfate (SLS) application to induce erythema, followed by the application of the test products. Instrumental evaluations included. The study included a known skin regenerator as a positive control and no product application as a negative control for comparative purposes. Instrumental evaluations were performed to assess various parameters of skin health and recovery. Skin color, specifically the erythema index, was measured using the Mexameter MX 18, which utilizes hemoglobin absorption wavelengths to quantify redness levels. Skin microcirculation was assessed through laser Doppler flowmetry (LDF), a technique that measures blood perfusion, providing insights into inflammatory and vascular responses. Additionally, transepidermal water loss (TEWL) was evaluated with the Tewameter TM 300, which applies Fick’s law to determine the integrity and functionality of the skin barrier. These precise measurements ensured a comprehensive understanding of the formulations’ effects on skin recovery and health.

Measurements were taken at baseline, post-SLS application and throughout the study. Statistical analyses, including the Wilcoxon signed-rank test, were performed with SPSS 23, setting significance at *p* < 0.05.

Regarding the F2CAA formulation and the corresponding cream, the inclusion criteria included women aged 50–65 years with visible signs of aging such as wrinkles, lack of firmness, or spots. Evaluations were conducted at baseline (D0), after 28 days of product application (D28), and at intermediary points for select tests. Instrumental efficacy was assessed using advanced technologies, including VISIA-CA System for UV, red, and visible spot analysis through standardized imaging; AEVA-HE System for 3D topography evaluation of skin roughness and wrinkles; Tewameter TM 300 for trans epidermal water loss (TEWL) measurements, assessing skin barrier function; Cutometer Dual MPA 580 for evaluating skin firmness and elasticity via negative pressure deformation; Corneometer CM825 for hydration assessment through capacitance measurement; Minolta Chromameter CR-400 for antioxidant efficacy by monitoring β-carotene oxidation; histamine test with the Perilont iontophoresis system to evaluate soothing; and inflammatory responses through laser Doppler flowmetry. Statistical evaluations were performed using appropriate paired and unpaired tests, depending on the variable type and measurement points. A two-tailed Student’s *t*-test was applied for parametric data to compare baseline (D0) and post-application (D28) results. For non-parametric data, the Wilcoxon signed-rank test was employed. The statistical significance threshold was set at *p* < 0.05.

Regarding the F3TAC formulation, participants included 14 individuals (12 completing the study) aged 18 to 25 years with mixed oily and oily skin exhibiting comedones or non-inflammatory acne. Both genders were represented, ensuring a diverse study population relevant to the product’s target users. The product was applied twice daily for 28 days. Measurements were taken at baseline (D0) and post-treatment (D28) to evaluate changes over the study period. Instrumental measurements were conducted to comprehensively evaluate the skin’s response to the treatment. A Sebumeter was used to measure the skin’s lipidic index, providing an assessment of sebum levels. The Corneometer CM825 evaluated skin hydration through capacitance-based measurements, offering insights into moisture levels. Trans epidermal water loss (TEWL) was assessed using the Tewameter TM 300, which measured the skin barrier’s integrity by quantifying water loss. Additionally, the VISIA System captured high-resolution images to analyze various skin parameters, including red and visible spots, pores and porphyrins, providing a detailed visualization of the skin’s condition before and after the treatment.

Data were analyzed using SPSS 23.0 software. Paired t-tests were applied to parametric data, and Wilcoxon signed-rank tests were used for non-parametric variables. Statistical significance was set at *p* < 0.05. Results were presented as mean percentage changes with associated *p*-values to validate the findings.

## 4. Conclusions

This study evaluates the potential of pCBs for skin health, highlighting their role in modulating the ECS, inflammation, and structural processes, with promising implications for cosmeceutical applications and skin disease treatments. Cannabinoids such as CBD, CBG and CBC are known to influence critical skin biological processes through ECS interactions. However, their effects, individually and in combination, under stress conditions remain insufficiently understood.

The study compares the effects of four cannabinoid-based formulations—F1CR2 (CBD + CBG), F1CR1 (CBD + CBC), F2CAA (CBD + CBG + CBC) and F3TAC (CBC + CBG)—in pre-treatment and treatment scenarios using HaCaT and HDF cells. It focuses on their impact on pro-inflammatory and anti-inflammatory cytokine modulation, cannabinoid receptor expression, and structural gene regulation, providing insights into their potential to promote skin regeneration, reduce inflammation and maintain homeostasis.

This study confirms the safety and efficacy of pCB-based formulations for skin applications, highlighting their potential to enhance regeneration and structural processes. The findings underscore the promise of cannabis-based products in cosmetics and dermatology, meeting the rising demand for natural, effective skincare solutions and shaping the future of modern skincare and therapeutic approaches.

**Impact of the findings on skin regeneration, aging and acne control**

Based on the results of the formulations’ efficacy, a strategic approach can be formulated to address a range of skin health concerns, including skin regeneration, aging and acne control. Each formulation has unique strengths, and by utilizing them either individually or in combination, more targeted and effective solutions can be achieved.

**Skin regeneration**

F1CR1 and F1CR2 are cannabinoid-based formulations demonstrating complementary strengths in skin regeneration, inflammation control, and structural integrity, with skin regeneration emerging as a hallmark benefit. F1CR1 (CBD + CBC) shows broad anti-inflammatory effects, modulating key mediators like TNF-α and IL-6 across keratinocytes and fibroblasts. It excels in vivo, where it reduces redness, improves microcirculation, and accelerates recovery by addressing inflammation and enhancing epidermal and dermal repair. Its modulation of the ECS, including GPR55 and CNR2, further supports tissue regeneration and barrier function, making it effective for managing acute skin inflammation.

F1CR2 (CBD + CBG) focuses on long-term skin regeneration, excelling in fibroblast activation under oxidative stress. It upregulates structural genes such as EGFR, TIMP3 and FN1, promoting collagen production, ECM stability and elasticity. This makes it particularly effective for dermal repair and chronic skin conditions. F1CR2’s targeted action on fibroblasts highlights its suitability for deeper tissue repair, anti-aging applications and sustained structural support.

While in vitro studies favored F1CR2 for tissue repair, F1CR1 proved superior in vivo due to its interaction with vascularization, hydration and immune responses. Together, F1CR1 and F1CR2 provide a synergistic skincare solution: F1CR1 addresses daily inflammation and skin protection, while F1CR2 focuses on overnight regeneration and long-term structural support. Their combined actions offer a comprehensive approach to skin health, emphasizing the pivotal role of regeneration in achieving optimal skin quality and resilience.

**Skin aging**

F2CAA, a cannabinoid-based formulation combining CBD, CBG and CBC, exhibits multifaceted actions on skin health, with a hallmark focus on addressing aging. Its anti-inflammatory properties are evident through the downregulation of pro-inflammatory cytokines like TNF-α and the upregulation of IL-10 in keratinocytes, effectively balancing inflammation suppression with anti-inflammatory signaling. This dual action positions F2CAA as a potent treatment for inflammatory skin conditions while supporting tissue repair and immune balance.

In aging skin, F2CAA’s modulation of structural genes such as TIMP3, FN1 and ELN plays a critical role in maintaining elasticity, promoting collagen production, and preventing excessive fibrosis and scarring. TIMP3 upregulation preserves ECM integrity, crucial for skin resilience and regeneration, while FN1 and ELN actions further enhance wound healing and elasticity, mitigating age-related skin degradation.

F2CAA also influences cannabinoid receptors, with reductions in GPR55 and increases in CNR1 and CNR2 in keratinocytes, offering protective effects against oxidative stress. In fibroblasts, receptor modulation strengthens skin resilience, aiding in wound healing and preventing further damage.

F2CAA’s anti-aging potential is amplified when combined with F1CR1, which excels in mitigating inflammation and oxidative stress. Together, these formulations offer a comprehensive strategy for aging skin, targeting inflammation control, ECM remodeling, and tissue regeneration to promote healthier, more resilient skin.

F3TAC, a cannabinoid-based formulation combining CBC and CBG, demonstrates context-dependent effects on inflammation and skin repair, with a hallmark focus on acne control. By modulating key structural genes like ELN, FN1, EGFR, and TIMP3, F3TAC supports ECM remodeling and wound healing, essential for addressing inflammation and tissue repair in acne-prone skin. CBC’s role as a CB2 receptor activator and free radical scavenger, combined with CBG’s anti-inflammatory and antimicrobial properties, offers targeted benefits in reducing oxidative stress, inflammation and acne-causing bacteria.

**Acne control**

For acne control, F3TAC effectively targets the initial stages of acne formation. Its anti-inflammatory action includes reducing pro-inflammatory cytokines such as TNF-α and IL-6, while its antimicrobial properties help combat acne-related bacterial overgrowth. Although in vitro results demonstrate significant reductions in inflammation, in vivo findings reflect the complexity of skin’s environment, including factors like microbiome interactions and sebum production. Further studies (already ongoing by our team) on the skin microbiome and sebocytes will provide deeper insights into F3TAC’s role in regulating acne by modulating sebum production and maintaining skin balance.

The combined use of F3TAC and F1CR1 offers a comprehensive strategy for acne management. F3TAC addresses immediate inflammation and microbial factors, while F1CR1 reduces redness, accelerates skin recovery, and promotes long-term barrier repair and regeneration. Together, these formulations minimize scarring, prevent acne lesions from worsening, and maintain clear, healthy skin, providing a synergistic approach for managing acne-prone skin conditions.

The findings on the four formulations emphasize the importance of selecting the appropriate cannabinoid-based formulation according to the specific skin condition and therapeutic goals. The different effects of these formulations highlight the potential of pCB combinations in enhancing skin health through targeted modulation of inflammatory, regenerative and structural pathways. As research into pCB-based therapies continues to evolve, these formulations represent promising candidates for advancing the management of a wide range of dermatological conditions. The formulations can be applied either as standalone treatments or in synergistic combinations, depending on the specific skin health challenge being addressed. This targeted approach maximizes the strengths of each formulation to achieve optimal outcomes in skin care.

## Figures and Tables

**Table 1:** Effect of F1CR1, F1CR2, F2CAA and F3TAC formulations on the pathways suggested from the in vitro assays.

	pCBs			Pro-Inflammation		Anti-Inflammation		Structural		Cann-Receptor	
	CBD	CBG	CBC	PT	T	PT	T	PT	T	PT	T
**F1CR1**	** 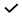 **		** 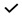 **	HaCaT and HDF: anti-inflammatory against OS HaCaT: protective against inflammation from TPA stress	HaCaT: complex regulatory action HDF: anti-inflammatory effect (dermis) HaCaT: no significant changes from TPA stress	HaCaT: no significant changes from OS HDF: complex modulation of inflammatory effect	HaCaT: modulation of inflammation and differentiation. HDF: anti-inflammatory action	HaCaT: modulation of ECM production and cell proliferation. HDF: prevent excessive collagen deposition, preventing scarring or fibrosis.	HaCaT: ECM remodeling protects against tissue breakdown and enhances wound healing.	HaCaT: anti-inflammatory action HDF: enhanced wound healing.	HaCaT modulation of the inflammatory action HDF: medium protective action
**F1CR2**	** 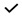 **	** 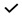 **		HaCaT: protective against OS, cellular repair. HDF: anti-inflammatory effect in dermal layer HaCaT: protective in reducing inflammation (TPA stress)	HaCaT and HDF: selective modulation of pro-inflammation and inflammation. HaCaT: targeted effect from inflammatory stimuli (TPA stress)	HaCaT: enhanced anti-inflammatory action HDF: complex modulation of anti-inflammatory signals in the dermis	HaCaT: inflammation modulation–shift HDF: anti-inflammatory response HaCaT: protective for inflammation (TPA-stress)	HaCaT: protection against ECM breakdown, reduced fibrosis and scarring. HDF: modulation of matrix production and cell proliferation.	HaCaT and HDF: potential reduction in fibrosis and scarring, protection against ECM degradation. HaCaT: tissue repair and protection against ECM degradation.	HaCaT: enhanced anti-inflammatory and antioxidant effect enhanced response to cannabinoid signaling and potential repair mechanisms. HDF: enhanced cannabinoid signaling	HDF: cellular proliferation and tissue remodeling.
**F2CAA**	** 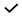 **	** 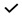 **	** 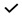 **	HaCaT: anti-inflammatory and regenerative action for OS and TPA stress. HDF: anti-inflammatory effect	HaCaT: complex modulation of inflammation HDF: anti-inflammatory effect HaCaT: selective modulation of inflammation	HaCaT: anti-inflammatory action for OS HDF: selective modulation of inflammation	HaCaT: modulation of inflammation and metabolic pathway. HDF: protection against OS HaCaT: protection against inflammation (TPA stress)	HaCaT: ECM protection and reduced fibrosis under OS. HDF: ECM production	HaCaT: protection against tissue breakdown HDF: complex modulation of ECM production and protection. HaCaT: tissue repair and protection (TPA stress).	HaCaT: enhanced anti-inflammatory signaling HDF: cell-specific modulation	HaCaT: modulate excessive signaling post-stress. HDF: protective effect
**F3TAC**		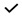	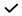	HaCaT: selective modulation of inflammatory pathways HDF: anti-inflammatory effect in dermal layer HaCaT anti-inflammatory action (TPA stress)	HaCaT: limited efficacy HDF: selective modulation of inflammatory responses HaCaT: selective modulation of inflammatory pathway	HaCaT: moderate anti-inflammatory support HDF: complex modulation of anti-inflammatory pathways	HaCaT: limited post-stress effects HDF: stronger impact on anti-inflammatory pathways HaCaT: no significant changes, stress-specific modulation (TPA stress).	HaCaT: enhanced ECM protection HDF: reduced ECM production and remodeling	HaCaT: enhanced protection against ECM degradation HDF: reduced ECM production HaCaT: enhanced ECM production and protection against degradation.	HaCaT: enhanced ECS activation HDF: protective mechanism	HaCaT: selective modulation HDF: enhanced ECS activation

HaCaT—human keratinocytes; HDF—human dermal fibroblasts; H_2_O_2_—hydrogen peroxide; TPA—phorbol 12-myrstate 13-acetate; OS: oxidative stress; ECM: extracellular matrix; PT—pre-treatment; T—treatment; CBD—cannabidiol; CBG—cannabigerol; and CBC—cannabichromene.

**Table 2:** Stress inducers tested for each human cell line.

Human Cell Line	Stress Inducers	Range of Concentrations
HaCaT	H_2_O_2_	50–400 μM
	TPA	5–20 μM
HDF	H_2_O_2_	50–400 μM

**Table 3:** Cannabinoid composition per formulation F1CR1, F1CR2, F2CAA and F3TAC.

Formulations and Creams	CBD	CBG	CBC
F1CR1	●		●
F1CR2	●	●	
F2CAA	●	●	●
F3TAC		●	●

**Table 4:** Set of primer sequences for the RT-qPCR.

Gene Name	Forward Primer	Reverse Primer
*IL-6*	ACCCCTGACCCAACCACAA	GTAGAGTAAGACGCGTCGAAA
*TNF-α*	TGAGGCCAAGCCCTGGTAT	AGTTAGCCGGGCTGATAGAG
*IL-36G*	CCACAAGGCATCTGCATGAG	AAGTCTCGAGTACGCGCAAT
*PPARγ*	AATCAAAGTGGAGCCTGCATCT	TTGAGGGAGTACCGTTAACTT
*IL-10*	GCATCTACAAAGCCATGAGTGAGT	TCTGTAGTCCCACCGCTGAGA
*GPR55*	GAGCATCAGCTTCTTCTTGCAAT	ACAGTAGTTTCTTAAGGCGTAC
*CNR1*	GTTTTGCCTGATGTGGACCAT	ACAAACGAGTCTGTAAAAGGGT
*CNR2*	GACAAGAAGTTTAGGCGGACACTAT	TAGGAGGGAGTTGTTCTACTCG
*ELN*	AAGCCTCAAAGCTGGATTCG	TGTAGACCCCGCGAAAAC
*FN1*	AGCCAACCAAGATGCAAATGT	GAATTCACAGACCGGGCGTT
*TIMP3*	TTCCCTGCGGAGTCGATAAA	TGACATCGCTTTGCGAAAGA
*EGFR*	GGCGTCCGCAAGTGTAAGAA	TCGTAGCATTTATGGAGAGTGAGTCT

## Data Availability

The data presented in this study are available on request from the corresponding authors due to commercial reasons. Restrictions apply to the availability of these data. Data were obtained from ExMceuticals and are available [from the authors/at URL] with the permission of EXMceuticals.

## Supplementary Materials

The following supporting information can be downloaded at: https://www.mdpi.com/article/10.3390/ijms252313142/s1.

## Abbreviations

2-AG—2-arachidonoylglycerol; AEA—anandamide; CB1—cannabinoid receptor type 1; CB2—cannabinoid receptor type 2; CNR1—gene that encodes cannabinoid receptor type 1; CNR2—gene that encodes cannabinoid receptor type 2; CBC—cannabichromene; CBD—cannabidiol; CBG—cannabigerol; CBN—cannabinol; ECM—extracellular matrix; ECS—endocannabinoid system; EGFR—epidermal growth factor receptor; ELN—elastin; FN1—fibronectin; GPR55—G protein-coupled receptor 55; H_2_O_2_—hydrogen peroxidase; HaCaT—human keratinocytes; HDF—human dermal fibroblast; IL-10—Interleukin 10; IL-36G—Interleukin-36 gamma; IL-6—Interleukin 6; MMPs—matrix metalloproteinases; NF-кB—nuclear factor kappa B; pCBs—phytocannabinoids; PKC—protein kinase C; PPARγ—proliferator-activated receptor gamma; ROS—reactive oxygen species; TEWL—trans epidermal water loss; THC—Δ9-tetrahydrocannabinol; TIMP3—metallopeptidase inhibitor 3; TNF-α—tumor necrosis factor alpha; TPA—phorbol 12-myrstate 13-acetate.
