# Supplementary material for: Exploring the Biological Activity of Phytocannabinoid Formulations for Skin Health Care: A Special Focus on Molecular Pathways
Source: Int J Mol Sci. 2024 Dec 6;25(23):13142. doi: 10.3390/ijms252313142 (PMC11641943; doi:10.3390/ijms252313142)
Supplement: Supplementary file 1 [file ijms-25-13142-s001.zip › ijms-3292151-supplementary.pdf]

Supplementary Materials

Exploring the Biological Activity of Phytocannabinoids Formulations for Skin Health Care: A Special Focus on Molecular Pathways

Guilherme Trigo [1], Mariana Coelho [2], Carolina Borges Ferreira [1], Matteo Melosini [1], Inês Sousa Lehmman [1], Catarina P. Reis [2,3,*], Maria Manuela Gaspar [2,3,*] and Susana Santos [1]

[1] R&D&I Department EXMceuticals Portugal Lda, 1749-016 Lisboa, Portugal; guilherme@tamartech.com (G.T.); carolina@tamartech.com (C.F.); matteo@tamartech.com (M.M.); ines@tamartech.com (I.L.); susana@tamartech.com (S.S)

[2] Research Institute for Medicines (iMed.ULisboa), Faculty of Pharmacy, Universidade de Lisboa, Avenida Professor Gama Pinto, 1649-003 Lisboa, Portugal mariana.coelho@ff.ulisboa.pt

[3] Institute of Biophysics and Biomedical Engineering (IBEB), Faculty of Sciences, Universidade de Lisboa, Campo Grande, 1749-016 Lisboa, Portugal

* Correspondence: catarinareis@ff.ulisboa.pt (C.P.R.); mgaspar@ff.ulisboa.pt (M.M.G.)

S1—Effect of formulations on pro-inflammatory markers

- **F1CR1**

In **HaCaT** cells exposed to **H_2O_2** stress, **F1CR1 pre-treatment** led to downregulation of IL-6 and strong downregulation of TNF-α and IL-36G, with IL-36G returning to basal levels. This response indicates that F1CR1 may effectively mitigate the pro-inflammatory cascade typically triggered by oxidative stress in keratinocytes. The reduction in these cytokines suggests a diminishing of the inflammatory response, which could be beneficial in preventing excessive inflammation in the epidermis.

Similarly, in **HDF** cells subjected to **H_2O_2** stress, **F1CR1 pre-treatment** resulted in relevant downregulation of IL-6, TNF-α, and IL-36G. This consistent downregulation across different cell types underscores the potential of F1CR1 to modulate inflammatory responses in various skin cell populations, suggesting a broader anti-inflammatory effect in the dermis as well.

When **HaCaT** cells were exposed to **TPA**, a known inflammatory agent, **F1CR1 pre-treatment** led to significant downregulation of IL-6, TNF-α, and IL-36G. This robust anti-inflammatory response against a direct inflammatory stimulus further supports the protective potential of F1CR1 against various inflammatory triggers in the skin. IL-6, TNF-α, and IL-36G are crucial pro-inflammatory cytokines involved in initiating and sustaining inflammatory responses in the skin. Their downregulation by F1CR1 pre-treatment suggests a potential mechanism for reducing skin inflammation. This modulation could be particularly beneficial in managing inflammatory skin conditions or in protecting the skin against environmental stressors that induce inflammation.

The results of **pre-treatment** with **F1CR1** followed by exposure to **H_2O_2** and **TPA** stressors demonstrate significant modulation of key pro-inflammatory cytokines in both HaCaT and HDF. This modulation suggests a potential protective effect of F1CR1 against inflammation in skin cells. These findings collectively indicate that F1CR1 may have significant potential as a preventive treatment for skin inflammation, capable of priming skin cells to better withstand both oxidative and direct inflammatory challenges.

In **HaCaT** cells **treated** with **F1CR1** after **H_2O_2** stress, a significant downregulation of IL-6 and TNF-α was observed, along with a small upregulation of IL-36G. IL-6 and TNF-α are potent pro-inflammatory cytokines that play crucial roles in initiating and sustaining inflammatory responses in the skin. Their downregulation suggests that F1CR1 may effectively mitigate the inflammatory cascade triggered by oxidative stress in keratinocytes. The slight upregulation of IL-36G, another pro-inflammatory cytokine, is interesting and may indicate a complex regulatory response that warrants further investigation.

In **HDF** cells **treated** with **F1CR1** after **H_2O_2** stress, a strong downregulation of both IL-6 and TNF-α was observed. This consistent downregulation across different cell types underscores the potential of F1CR1 to modulate inflammatory responses in various skin cell populations. The strong anti-inflammatory effect in fibroblasts suggests that F1CR1 may be particularly effective in reducing inflammation in the dermal layer of the skin.

Interestingly, when **HaCaT** cells were **treated** with **F1CR1** after **TPA** stress, no significant changes were observed in IL-6, TNF-α, or IL-36G levels. This difference in response between H_2O_2 and TPA stress highlights the specificity of F1CR1's effects and the importance of the type of stressor in determining cellular responses.

- **F1CR2**

When **HaCaT** cells were **pre-treated** with **F1CR2** before exposure to **H_2O_2-induced** oxidative stress, an upregulation of IL-6 and a downregulation of TNF-α and IL-36G were observed. This suggests that F1CR2 may have a protective or modulatory effect, potentially priming HaCaT cells to better manage oxidative stress by altering the inflammatory response. The downregulation of TNF-α and IL-36G could indicate that F1CR2 either neutralized some of the oxidative effects of H_2O_2 or enhanced the cells' resilience, reducing the need for a strong inflammatory response.
In contrast, in **HDF** cells, **pre-treated** with **F1CR2** led to no significant changes in IL-6 levels, a strong downregulation of TNF-α, and an upregulation of IL-36G. This differential response suggests that F1CR2 induces a cell-type-specific modulation of cytokine expression. The upregulation of IL-36G in HDF cells implies that IL-36G may play a distinct role in these cells, possibly related to enhanced tissue repair or a more targeted inflammatory response necessary for fibroblast function in tissue regeneration. These findings highlight the potential of F1CR2 as a regenerative agent with anti-inflammatory properties, capable of tailoring its effects based on the specific cellular context.

When **HaCaT** were **pre-treated** with **F1CR2** before being exposed to **TPA**, there was a downregulation of the inflammatory cytokines IL-6, TNF-α, and a particularly strong downregulation of IL-36G. This outcome suggests that F1CR2 may have a protective or modulatory effect on the cells, likely "priming" them to be less reactive to the pro-inflammatory signals typically induced by TPA. F1CR2 might suppress the initial inflammatory cascade triggered by TPA, helping to control the extent of inflammation. Similar, the downregulation of TNF-α following F1CR2 pre-treatment suggests that F1CR2 may inhibit excessive immune cell recruitment and activation, reducing the overall inflammatory burden on the cells. This reduction could protect the skin from prolonged or chronic inflammation, which can lead to tissue damage and impaired regeneration. The strong downregulation of IL-36G after F1CR2 pre-treatment suggests that the formulation might effectively limit the amplification of the inflammatory response, preventing the cascade of events that typically follows chemical exposure. This downregulation is particularly relevant in the context of conditions like psoriasis, where IL-36G plays a critical role in driving skin inflammation and thickening.

For cells **treated** with **F1CR2** after being subjected to **H_2O_2** stress, a strong upregulation of IL-6, and downregulation of TNF-α in both **HaCaT** cells and **HDF** was observed. Regarding IL-36G, it was downregulated in HaCaT and notably upregulated in HDF cells. The strong upregulation of IL-6 in both HaCaT and HDF after F1CR2 treatment suggests that it may be enhancing the cells' ability to respond to oxidative damage. In this context, the increase in IL-6 could be beneficial, as it may help to promote a repair response following oxidative stress. This indicates that post-stress treatment with F1CR2 might not only manage inflammation but also stimulate regenerative processes necessary for skin recovery. The downregulation of TNF-α in both cell types suggests that F1CR2 is effectively reducing excessive inflammatory responses. By lowering TNF-α levels, F1CR2 may help prevent prolonged or chronic inflammation, thereby protecting the cells from further damage and promoting a controlled and beneficial inflammatory environment that supports healing rather than causing additional harm. This is in accordance with the downregulation of IL-36G in HaCaT indicating that F1CR2 may help to mitigate excessive inflammatory signaling. By reducing IL-36G levels, F1CR2 could help to prevent excessive or damaging inflammation in HaCaT, promoting a more balanced inflammatory response that supports regeneration without leading to unnecessary tissue damage. The notable upregulation of IL-36G in HDF suggests a different role for IL-36G when compared to HaCaT and may play a role in promoting tissue repair and remodeling. The upregulation of IL-36G could indicate that F1CR2 is enhancing the HDF´s ability to manage the post-stress environment, potentially by promoting processes that support wound healing and ECM production. This upregulation might reflect a need for a more targeted inflammatory response that aids in the regeneration of dermal tissues following oxidative damage, for which IL-36G may play a critical role in the later stages of the inflammatory or repair processes.

In contrast, when **HaCaT** cells were **treated** with **F1CR2,** after being exposed to **TPA**, an upregulation of IL-6, TNF-α, and a strong upregulation of IL-36G was observed. This suggests that once the inflammatory response has been initiated by TPA, applying F1CR2 might not be sufficient to counteract the strong pro-inflammatory signals already in motion. The increase in IL-6, TNF-α and IL-36G after F1CR2 treatment suggests that the cells remain highly reactive to inflammatory stimuli once the response has been triggered. This continued upregulation could indicate that F1CR2, when applied post-stress, interacts with the already activated pathways in a way that does not mitigate the inflammation.

- **F2CAA**

In **HaCaT** cells **pre-treated** with **F2CAA** and stressed with **H_2O_2**, an upregulation of IL-6 and a downregulation of TNF-α and IL-36G was observed. The upregulation of IL-6, typically considered pro-inflammatory, may indicate a compensatory mechanism.

In **HDF** cells **pre-treated** with **F2CAA** and exposed to **H_2O_2** stress, a consistent downregulation of all three pro-inflammatory cytokines was observed, with TNF-α showing the most pronounced decrease. This suggests a potentially broad anti-inflammatory effect of F2CAA in the dermal layer during. The significant downregulation of TNF-α, a key mediator of inflammation, is particularly noteworthy and may indicate a potent anti-inflammatory action of F2CAA in fibroblasts.

When **HaCaT** cells **pre-treated** with **F2CAA** were exposed to **TPA** it was denoted a significant downregulation across all three cytokines, with IL-6 returning to basal levels and IL-36G showing the most significant decrease. This strong anti-inflammatory response against a direct inflammatory stimulus underscores F2CAA's potential to prime keratinocytes against inflammatory stress.

Relative to **HaCaT** cells **treated** with **F2CAA,** after **H_2O_2** stress, an upregulation of IL-6 was observed, along with a downregulation of TNF-α. This mixed response suggests a complex modulation of the inflammatory cascade, where F2CAA may be selectively influencing different pro-inflammatory pathways. The downregulation of TNF-α suggests a potential anti-inflammatory effect of F2CAA in keratinocytes under oxidative stress.

In **HDF** cells **treated** with **F2CAA,** after **H_2O_2** stress, a similar pattern was observed, with upregulation of IL-6 and a strong downregulation of TNF-α. This consistent response across different cell types underscores the potential of F2CAA to modulate inflammatory responses in various skin cell populations. The strong downregulation of TNF-α in fibroblasts is particularly noteworthy, as it suggests a potent anti-inflammatory effect in the dermal layer when faced with oxidative stress.

When **HaCaT** cells were **treated** with **F2CAA,** after **TPA**-induced stress, a slight upregulation of IL-6 and TNF-α was observed, along with a strong downregulation of IL-36G. This response to inflammatory stress differs from the oxidative stress response, highlighting the context-dependent effects of F2CAA. The strong downregulation of IL-36G, a pro-inflammatory cytokine involved in various skin inflammatory conditions, suggests that F2CAA may have particular efficacy in modulating specific inflammatory pathways triggered by TPA.

- **F3TAC**

In **HaCaT** cells pre-treated with **F3TAC,** before **H_2O_2** stress, a strong upregulation of IL-6 was observed, along with downregulation of TNF-α and IL-36G. This integrated response suggests a complex modulation of the inflammatory cascade, where F3TAC may be selectively influencing different pro-inflammatory pathways. The upregulation of IL-6 may indicate a compensatory mechanism or a potential protective role in this context.

In **HDF pre-treated** with **F3TAC,** before **H_2O_2** stress, a consistent downregulation of all three pro-inflammatory cytokines was observed, with TNF-α showing the most pronounced decrease. This suggests a potentially broad anti-inflammatory effect of F3TAC in the dermal layer during oxidative stress. The significant downregulation of TNF-α, a key mediator of inflammation, is particularly noteworthy and may indicate a potent anti-inflammatory action of F3TAC in fibroblasts.

For **HaCaT** cells **pre-treated** with **F3TAC,** before **TPA** stress, a significant downregulation across all three cytokines was observed, with IL-36G showing the most significant decrease. This strong anti-inflammatory response against a direct inflammatory stimulus underscores F3TAC's potential to prime keratinocytes against inflammatory stress.

When **HaCaT** cells were **treated** with **F3TAC,** after **H_2O_2** stress, no significant changes were observed in TNF-α, IL-6, and IL-36G levels. This suggests that F3TAC may have limited efficacy in modulating these pro-inflammatory cytokines when applied after oxidative stress has occurred in keratinocytes.

In **HDF** cells treated with **F3TAC,** after **H_2O_2** stress, an upregulation of IL-6 was observed, along with a strong downregulation of TNF-α and no significant changes in IL-36G. This diverse response indicates that F3TAC may have different effects on various pro-inflammatory pathways in fibroblasts when applied after oxidative stress.

HaCaT cells **treated** with **F3TAC,** after **TPA** stress, showed a slight upregulation of IL-6 and a strong downregulation of IL-36G. This response to inflammatory stress differs from the oxidative stress response, highlighting the context-dependent effects of F3TAC. The strong downregulation of IL-36G, a pro-inflammatory cytokine involved in various skin inflammatory conditions, suggests that F3TAC may have particular efficacy in modulating specific inflammatory pathways triggered by TPA.

S2—Effect of formulations on anti-inflammatory markers

- **F1CR1**

In **HaCaT** cells **pre-treated** with **F1CR1**, and subjected to $\mathbf{H_2O_2}$ stress, no significant changes were observed in the regulation of IL-10 and PPARγ. This suggests that F1CR1 pre-treatment may not significantly alter the anti-inflammatory response mediated by these factors in keratinocytes under oxidative stress conditions.
However, in **HDF** cells **pre-treated** with **F1CR1** and exposed to $\mathbf{H_2O_2}$ stress, a relevant downregulation of both IL-10 and PPARγ was observed. IL-10 is a potent anti-inflammatory cytokine, while PPARγ is a nuclear receptor with anti-inflammatory properties. Their downregulation in fibroblasts might indicate a complex modulation of the inflammatory response, possibly suggesting that F1CR1 pre-treatment alters the cell's anti-inflammatory mechanisms in response to oxidative stress.
Interestingly, when **HaCaT** cells were **pre-treated** with **F1CR1** and subjected to **TPA** stress, a significant downregulation of both IL-10 and PPARγ was observed. TPA is known to induce inflammation, and this downregulation of anti-inflammatory factors might seem counter intuitive. However, it could indicate a complex regulatory response where F1CR1 pre-treatment modulates the inflammatory pathway through other mechanisms.

In **HaCaT** cells **treated** with **F1CR1,** after $\mathbf{H_2O_2}$ stress, a downregulation of PPARγ was observed. PPARγ is known to play a role in regulating inflammation and cell differentiation. Its downregulation in this context might suggest a complex modulation of the cellular stress response, potentially affecting inflammatory pathways and keratinocyte differentiation.
In **HDF** cells **treated** with **F1CR1,** after $\mathbf{H_2O_2}$ stress, an upregulation of IL-10 and a strong downregulation of PPARγ were observed. IL-10 is a potent anti-inflammatory cytokine, and its upregulation suggests that F1CR1 may be promoting an anti-inflammatory response in fibroblasts under oxidative stress. The strong downregulation of PPARγ in fibroblasts, similar to that seen in keratinocytes, further indicates a significant modulation of cellular responses to oxidative stress, potentially affecting both inflammatory and metabolic pathways.
Regarding **HaCaT** cells **treated** with **F1CR1,** after **TPA**-induced stress, a downregulation of IL-10 was observed, with no significant changes in PPARγ expression. This response to inflammatory stress differs from the oxidative stress response, suggesting that F1CR1's effects are stress-specific. The downregulation of IL-10, an anti-inflammatory cytokine, in response to TPA might indicate a complex regulation of the inflammatory cascade, potentially involving other compensatory mechanisms.

- **F1CR2**

In this assay, when **HaCat** cells were **pre-treated** with **F1CR2**, it was observed a strong downregulation of IL-10 and a strong upregulation of PPARγ. Similarly, in **HDF** cells, **pre-treatment** led to a downregulation of IL-10 and a strong upregulation of PPARγ. The strong downregulation of IL-10 observed in HaCaT cells suggests that pre-treatment with F1CR2, in the context of subsequent oxidative stress from H_2O_2, may diminish the cell's production of this anti-inflammatory cytokine. This could indicate that F1CR2 primes the cells to manage oxidative stress more directly, possibly by shifting the effect away from an anti-inflammatory response towards other mechanisms, such as stress tolerance or repair processes. The reduced need for IL-10 might imply that the pre-treatment helps reduce the overall inflammatory burden, making IL-10 less necessary. The strong upregulation of PPARγ in HaCaT cells following pre-treatment with F1CR2 indicates a significant shift towards promoting anti-inflammatory responses and possibly enhancing cellular repair and differentiation processes. PPARγ activation might be part of a broader strategy by which the cells enhance their metabolic strength and recovery potential in response to oxidative stress. This suggests that F1CR2 primes the cells for an adaptive response that prioritizes metabolic reprogramming and anti-inflammatory regulation through PPARγ pathways.

Similar to HaCaT cells, the downregulation of IL-10 in **HDF** cells suggests that **F1CR2 pre-treatment** reduces the effect on this anti-inflammatory pathway, potentially due to an enhancement of other protective mechanisms. This response might indicate that HDF cells, like HaCaT cells, are being primed to handle oxidative stress with less dependency on IL-10-mediated anti-inflammatory actions.

The strong upregulation of PPARγ in **HDF** cells suggests that similar to HaCaT cells, the **F1CR2 pre-treatment** promotes an adaptive response focused on enhancing anti-inflammatory and metabolic regulatory processes. PPARγ role in lipid metabolism and inflammation modulation may be particularly relevant in fibroblasts, which are key players in tissue repair and ECM production. This upregulation indicates that F1CR2 might be promoting a cellular environment conducive to healing and inflammation resolution.

In contrast, when **F1CR2** was applied as **treatment**, after H_2O_2-induced stress, the results varied. In HaCaT cells, no significant changes were observed in IL-10 levels, but PPARγ was still upregulated. The lack of significant change in IL-10 levels in HaCaT cells suggests that treatment with F1CR2 does not further modulate the anti-inflammatory response mediated by IL-10. This could imply that once the oxidative stress response has been initiated, the cellular regulation of IL-10 is already set, and F1CR2 may not significantly influence this pathway under these conditions. By its turn, the continued upregulation of PPARγ even after stress indicates that F1CR2 treatment promotes the activation of this regulatory pathway, regardless of whether it is applied before or after the oxidative stress. This suggests that PPARγ activation is a consistent effect of F1CR2 treatment, likely contributing to enhanced anti-inflammatory responses and metabolic adjustments necessary for recovery from oxidative damage.

Unlike in HaCaT cells, **HDF** cells showed an upregulation of IL-10 after **treatment** with **F1CR2** post-H_2O_2 stress. This suggests a differential response in fibroblasts, where IL-10 may play a more active role in managing inflammation and promoting tissue repair following oxidative damage. The increase in IL-10 could indicate that, in HDF, F1CR2 supports the resolution phase of inflammation by enhancing anti-inflammatory signaling when applied after the stressor has already triggered an inflammatory response. Similar to HaCaT cells, PPARγ is upregulated in HDF cells after treatment with F1CR2, reinforcing the idea that F1CR2 consistently promotes PPARγ-mediated pathways in response to oxidative stress. This upregulation further supports the notion that PPARγ is crucial in modulating the inflammatory response and promoting cell survival and repair processes in the context of oxidative damage.

- **F2CAA**

In **HaCaT** cells **pre-treated** with **F2CAA** and **stressed** with **H_2O_2**, a slight upregulation of IL-10 was observed, while PPARγ showed no significant changes. IL-10 is a potent anti-inflammatory cytokine, and its upregulation suggests that F2CAA may be enhancing the keratinocytes' ability to counteract oxidative stress-induced inflammation. The maintenance of PPARγ levels indicates that the anti-inflammatory and metabolic functions associated with this nuclear receptor remain stable under these conditions.

In **HDF** cells **pre-treated** with **F2CAA** and exposed to **H_2O_2** stress, a relevant downregulation of both IL-10 and PPARγ was observed. This response in fibroblasts differs from that in keratinocytes, highlighting the cell type-specific effects of F2CAA. The downregulation of IL-10 and PPARγ in fibroblasts might indicate a complex modulation of the inflammatory response, potentially altering the balance between pro- and anti-inflammatory signals in the dermal layer under oxidative stress.

When **HaCaT** cells **pre-treated** with **F2CAA** were exposed to **TPA**, a significant downregulation of both IL-10 and PPARγ was observed. This response to inflammatory stress differs from the oxidative stress response, suggesting that F2CAA's effects are stress-specific. The downregulation of these anti-inflammatory mediators in response to TPA might indicate a complex regulation of the inflammatory cascade, potentially involving other compensatory mechanisms.

In **HaCaT** cells **treated** with **F2CAA** after **H_2O_2** stress, a strong downregulation of both PPARγ and IL-10 was observed. This response suggests that F2CAA may be modulating the inflammatory and metabolic pathways in keratinocytes under oxidative stress conditions.

In **HDF** cells **treated** with **F2CAA** after **H_2O_2** stress, a different pattern emerged. A downregulation of IL-10 was observed, similar to HaCaT cells, but PPARγ was upregulated. The upregulation of PPARγ in fibroblasts might suggest a protective mechanism against oxidative stress, potentially promoting anti-inflammatory and antioxidant responses in the dermal layer.

For **HaCaT** cells **treated** with **F2CAA** after **TPA**-induced stress, an upregulation of both IL-10 and PPARγ was observed. This response to inflammatory stress differs markedly from the oxidative stress response. The upregulation of these anti-inflammatory mediators might indicate a protective mechanism against TPA-induced inflammation in keratinocytes.

- **F3TAC**

In **HaCaT pre-treated** with **F3TAC,** before **H_2O_2** stress, a slight upregulation of IL-10 was observed, while PPARγ showed no significant changes. This suggests a potential enhancement of anti-inflammatory responses in keratinocytes under oxidative stress conditions. The maintenance of PPARγ levels indicates that the metabolic functions associated with this nuclear receptor remain stable.

In **HDF** pre-treated with **F3TAC,** before **H_2O_2** stress, a relevant downregulation of both IL-10 and PPARγ was observed. This response in fibroblasts differs from that in keratinocytes, highlighting once again the cell type-specific effects of F3TAC. The downregulation of these anti-inflammatory mediators in fibroblasts might indicate a complex modulation of the inflammatory response, potentially altering the balance between pro- and anti-inflammatory signals in the dermal layer under oxidative stress.

For **HaCaT** cells **pre-treated** with **F3TAC,** before **TPA** stress, a significant downregulation of both IL-10 and PPARγ was observed. This response to inflammatory stress differs from the oxidative stress response, suggesting that F3TAC's effects are stress-specific. The downregulation of these anti-inflammatory mediators in response to TPA might indicate a complex regulation of the inflammatory cascade, potentially involving other compensatory mechanisms.

Regarding **HaCaT treatment** with **F3TAC,** after **H_2O_2** stress, a slight downregulation of both PPARγ and IL-10 was observed, though the changes were not significant. This suggests that F3TAC may have limited effects on these anti-inflammatory markers when applied after oxidative stress has occurred in keratinocytes.

In **HDF** cells **treated** with **F3TAC,** after **H_2O_2** stress, downregulation of both IL-10 and PPARγ was observed. This consistent response across different treatment scenarios in fibroblasts suggests that F3TAC may have a more pronounced effect on modulating anti-inflammatory pathways in the dermal layer, regardless of whether it is applied before or after stress induction.

HaCaT cells **treated** with **F3TAC,** after **TPA** stress, showed no significant changes in IL-10 and PPARγ levels. This lack of response to inflammatory stress differs from both the pre-treatment scenario and the oxidative stress response, highlighting the context-dependent effects of F3TAC.

S3—Effect of formulations on structural markers

- **F1CR1**

In HaCaT cells **pre-treated** with **F1CR1** and subjected to **H_2O_2** stress, a strong downregulation of FN1 was observed, along with an upregulation of EGFR and a stronger upregulation of TIMP3. This pattern suggests that F1CR1 may be modulating the ECM production and cell proliferation in response to oxidative stress. The downregulation of FN1 could indicate a reduction in fibrosis, while the upregulation of EGFR might promote cell survival and proliferation. The strong upregulation of TIMP3 suggests enhanced regulation of ECM remodeling, which may protect against oxidative damage-induced tissue breakdown.

In **HDF** cells **pre-treated** with **F1CR1** and exposed to **H_2O_2** stress, a downregulation was observed for ELN, FN1, and TIMP3. This response in fibroblasts differs from that in keratinocytes, highlighting the cell type-specific effects of F1CR1. The downregulation of ELN and FN1 might indicate a modulation of the ECM production in response to oxidative stress, potentially preventing excessive fibrosis. The downregulation of TIMP3 in this context is intriguing and may suggest a complex regulation of matrix remodeling in fibroblasts under oxidative stress conditions.

When **HaCaT** cells were **pre-treated** with **F1CR1** and subjected to **TPA**-induced stress, a downregulation was observed for ELN, FN1, TIMP3, and EGFR. This response to inflammatory stress differs from the oxidative stress response, suggesting that F1CR1's effects are also stress specific. The downregulation of these genes might indicate a protective mechanism against excessive inflammation and proliferation typically induced by TPA.

In **HaCaT** cells **treated** with **F1CR1,** after **H_2O_2** stress, the downregulation of FN1 suggests a potential modulation of ECM production, which may help prevent excessive fibrosis in response to oxidative stress. The upregulation of EGFR (epidermal growth factor receptor) to basal levels indicates a restoration of normal cell proliferation and differentiation signaling. The stronger upregulation of TIMP3 suggests enhanced regulation of ECM remodeling, which may protect against oxidative damage-induced tissue breakdown.

In **HDF** cells **treated** with **F1CR1,** after **H_2O_2** stress, the slight upregulation of ELN might indicate a mild protective response to maintain skin elasticity. The slight downregulation of FN1, EGFR, and TIMP3 suggests a more subtle modulation of ECM production, cell proliferation, and matrix remodeling in fibroblasts compared to keratinocytes.

When **HaCaT** cells were **treated** with **F1CR1,** after **TPA**-induced stress, no significant changes were observed for ELN, TIMP3, and EGFR, while FN1 was upregulated. This response to inflammatory stress differs from the oxidative stress response, suggesting that F1CR1's effects are stress specific. The upregulation of FN1 might indicate an enhanced wound healing response to inflammatory stimuli.

- **F1CR2**

In **HaCaT** cells **pre-treated** with **F1CR2** and subjected to **H_2O_2** stress, the downregulation of ELN suggests a potential protective mechanism against excessive elastin degradation often associated with oxidative damage. The strong downregulation of FN1 indicates a modulation of ECM production, which may help prevent excessive fibrosis in response to oxidative stress. Conversely, the strong upregulation of EGFR suggests activation of proliferative and survival pathways, potentially enhancing the cells' ability to cope with oxidative stress. The strong upregulation of TIMP3 indicates enhanced regulation of ECM remodeling, which may protect against oxidative damage-induced tissue breakdown.

In **HDF** cells **pre-treated** with **F1CR2** and subjected to **H_2O_2** stress, a different response is observed. The strong upregulation of both ELN and FN1 suggests a protective mechanism aimed at enhancing structural support and wound healing capacity in the face of oxidative stress. The slight downregulation of TIMP3 may indicate a slight shift towards matrix remodeling, possibly to accommodate the increased production of structural proteins.

For **HaCaT** cells **pre-treated** with **F1CR2** and subjected to **TPA**-induced stress, the downregulation of ELN and EGFR indicates a shift towards reduced proliferation and structural protein production. This response could be a mechanism to counteract the pro-inflammatory effects of TPA, potentially mitigating excessive inflammatory responses in the skin.

In **HaCaT** cells **treated** with **F1CR2,** after **H_2O_2** stress, the strong downregulation of ELN suggests a potential protective mechanism against excessive ELN degradation often associated with oxidative damage. The upregulation of FN1 indicates enhanced ECM production, which may promote wound healing and tissue repair. The strong upregulation of EGFR and TIMP3 suggests activation of proliferative pathways and regulation of ECM remodeling, respectively. These changes collectively point to a coordinated response aimed at tissue regeneration and protection against oxidative damage.

In contrast, **HDF** cells **treated** with **F1CR2,** after **H_2O_2** stress showed strong downregulation of both ELN and FN1, indicating a different response in fibroblasts. This could represent a mechanism to prevent excessive scarring or fibrosis in response to oxidative stress. The small upregulation of TIMP3 suggests a more modest regulation of matrix remodeling compared to keratinocytes.

For **HaCaT** cells **treated** with **F1CR2,** after **TPA**-induced stress, the upregulation of ELN and TIMP3, coupled with a small downregulation of EGFR, indicates a shift towards structural support and matrix stabilization, potentially counteracting the pro-inflammatory effects of TPA.

- **F2CAA**

In **HaCaT** cells **pre-treated** with **F2CAA,** before **H_2O_2** stress, a strong downregulation of FN1 and EGFR was observed, coupled with a very strong upregulation of TIMP3. This suggests a potential protective mechanism against oxidative stress-induced inflammation and excessive ECM degradation. The downregulation of FN1 may indicate a reduction in fibrosis-related processes, while increased TIMP3 could enhance protection against ECM breakdown.

In **HDF** cells, **F2CAA pre-treatment,** before **H_2O_2** stress, led to a downregulation of ELN and FN1, upregulation of EGFR, and no significant changes in TIMP3. This differential response in fibroblasts highlights the cell type-specific effects of F2CAA and may indicate a modulation of ECM production and cell proliferation in the dermal layer.

For **HaCaT** cells **pre-treated** with **F2CAA,** before **TPA** stress, strong downregulation of ELN, FN1, TIMP3, and EGFR was observed. This distinct response to inflammatory stress suggests that F2CAA may modulate the inflammatory cascade differently depending on the type of stressor, potentially limiting excessive ECM production and cell proliferation in response to TPA-induced inflammation.

Regarding **HaCaT** cells **treatment** with **F2CAA,** after **H_2O_2** stress, a downregulation of ELN, FN1, and EGFR was observed, along with strong upregulation of TIMP3. This response suggests that F2CAA may help mitigate oxidative stress-induced damage by reducing excessive ECM production and cell proliferation while enhancing protection against ECM degradation. The strong upregulation of TIMP3 is particularly noteworthy, as it indicates enhanced protection against MMPs, which are often elevated during oxidative stress.

In **HDF** cells **treated** with **F2CAA,** after **H_2O_2** stress, downregulation of FN1, ELN and TIMP3 was observed. This response in fibroblasts differs from that in keratinocytes, highlighting the cell type-specific effects of F2CAA. The downregulation of these ECM components in fibroblasts might indicate a complex modulation of the stress response, potentially altering the balance between ECM production and degradation in the dermal layer under oxidative stress conditions.

HaCaT cells **treated** with **F2CAA,** after **TPA stress,** showed slight upregulation of ELN, upregulation of FN1 and TIMP3, and slight downregulation of EGFR. This response to inflammatory stress differs from the oxidative stress response, suggesting that F2CAA's effects are stress-specific. The upregulation of ECM components (ELN and FN1) along with TIMP3 suggests that **F2CAA** may promote tissue repair and limit excessive ECM degradation in response to TPA-induced inflammation. The slight downregulation of EGFR indicates a potential moderation of cell proliferation, which could be beneficial in controlling excessive inflammatory responses.

- **F3TAC**

In **HaCaT pre-treated** with **F3TAC,** before **H_2O_2** stress, upregulation of ELN suggests enhanced skin elasticity and resilience. Strong downregulation of FN1 and EGFR may indicate reduced cell proliferation and migration. Very strong upregulation of TIMP3 suggests enhanced protection against ECM degradation.

In **HDF pre-treated** with **F3TAC,** before **H_2O_2** stress, downregulation of ELN, FN1, and TIMP3 indicates reduced ECM production and remodeling. No significant changes in EGFR suggest maintained cell proliferation capacity.

For **HaCaT** cells **pre-treated** with **F3TAC,** before **TPA** stress, strong downregulation of ELN and TIMP3, along with downregulation of FN1 and EGFR, suggest a significant reduction in ECM production and cell proliferation in response to inflammatory stress.

When **HaCaT** cells **were** treated with **F3TAC,** after **H_2O_2** stress, no significant changes in FN1 indicate maintained ECM production. Strong downregulation of EGFR suggests reduced cell proliferation. Strong upregulation of TIMP3 indicates enhanced protection against matrix degradation.

In **HDF** cells **treated** with **F3TAC,** after **H_2O_2** stress, downregulation of ELN, FN1, and TIMP3 suggests reduced ECM production and remodeling. No significant changes in EGFR indicate maintained cell proliferation capacity.

HaCaT cells **treated** with **F3TAC,** after **TPA** stress, showed upregulation of ELN, TIMP3, and slight upregulation of FN1, suggesting enhanced ECM production and protection. No changes in EGFR indicate maintained cell proliferation capacity.

S4—Effect of the formulations on cannabinoid receptor markers

- **F1CR1**

In **HaCaT** cells **pre-treated** with **F1CR1** and subjected to **H_2O_2** stress, a downregulation of GPR55 and CNR2 was observed, with no significant changes in CNR1 expression. The downregulation of GPR55, which is often associated with pro-inflammatory effects, could indicate a potential anti-inflammatory action of F1CR1. The reduction in CNR2, typically involved in immune modulation, might reflect a complex regulatory response to oxidative stress.

In **HDF** cells **pre-treated** with **F1CR1,** and exposed to **H_2O_2** stress, a different pattern emerged. An upregulation of GPR55 was observed, along with a significant downregulation of CNR2, while CNR1 remained unchanged. This contrasting response in fibroblasts highlights the cell type-specific effects of F1CR1. The upregulation of GPR55 in fibroblasts might be related to its role in cell proliferation and migration, potentially promoting wound healing responses under oxidative stress conditions.

When **HaCaT** cells were **pre-treated** with **F1CR1** and subjected to **TPA**-induced stress, an upregulation of GPR55 was observed, along with a significant downregulation of CNR2, while CNR1 remained unchanged. This response to inflammatory stress differs from the oxidative stress response in keratinocytes, suggesting that F1CR1's effects are also stress-specific. The consistent downregulation of CNR2 across different cell types and stress conditions is particularly noteworthy. CNR2 is known for its role in modulating immune responses and inflammation in the skin. Its downregulation might indicate a complex modulation of the inflammatory response by F1CR1, potentially preparing the cells to respond differently to subsequent stressors. The lack of significant changes in CNR1 expression across all conditions suggests that F1CR1's effects may be primarily mediated through other cannabinoid receptors or pathways. These results highlight the complex and context-dependent effects of F1CR1 on the ECS in skin cells.

Regarding **HaCaT** cells **treated** with **F1CR1,** after **H_2O_2** stress, no significant changes were observed for GPR55, while CNR2 was upregulated and CNR1 was downregulated. This pattern suggests that F1CR1 may be modulating the inflammatory response in keratinocytes under oxidative stress conditions. The upregulation of CNR2, which is often associated with anti-inflammatory effects, could indicate a protective mechanism against oxidative damage. The downregulation of CNR1 might reflect a complex regulatory response to balance proliferation and differentiation in stressed keratinocytes.

In **HDF** cells **treated** with **F1CR1,** after **H_2O_2** stress, a slight upregulation was observed for both GPR55 and CNR1. This subtle increase in receptor expression might suggest a mild activation of proliferative and protective pathways in fibroblasts under oxidative stress. The upregulation of CNR1 could potentially promote cell survival and reduce inflammation in the dermal layer.

By its turn, when **HaCaT** cells were **treated** with **F1CR1,** after **TPA**-induced stress, a significant upregulation was observed for all three receptors: GPR55, CNR2, and CNR1. This robust response to inflammatory stress differs markedly from the oxidative stress response, suggesting that F1CR1's effects are stress-specific. The upregulation of all three receptors might indicate a comprehensive activation of the ECS to decrease inflammation and promote regeneration in keratinocytes exposed to TPA.

- **F1CR2**

The application of **F1CR2** prior to **H_2O_2-**induced oxidative stress in HaCaT cells resulted in significant changes in the expression of the cannabinoid receptors. Notably, there was a downregulation of GPR55 and CNR2, accompanied by an upregulation of CNR1.

When **HaCat** cells were **treated** with **F1CR2,** after the **H_2O_2-**induced oxidative stress. The results demonstrated a persistent downregulation of GPR55 in HaCaT cells following F1CR2 treatment and H_2O_2 stress. However, the strong downregulation of both CNR1 and CNR2 post-stress was observed. In contrast to HaCaT cells, HDFs exhibited a strong upregulation of GPR55 and CNR2, coupled with a downregulation of CNR1.

- **F2CAA**

In **HaCaT pre-treated** with **F2CAA,** before **H_2O_2** stress, GPR55 was downregulated while CNR2 and CNR1 were upregulated. This suggests a potential protective mechanism against oxidative stress, as CNR1 and CNR2 activation is generally associated with anti-inflammatory and antioxidant effects. The downregulation of GPR55, which can promote inflammation when activated, further supports this protective role.

In **HDF**, **F2CAA pre-treatment,** before **H_2O_2** stress, led to significant downregulation of GPR55 and CNR2, with upregulation of CNR1. This differential response in fibroblasts highlights the cell type-specific effects of F2CAA and may indicate a tailored protective mechanism in the dermal layer.

For **HaCaT** cells **pre-treated** with **F2CAA,** before **TPA** stress, GPR55 was upregulated, CNR2 was significantly downregulated, and CNR1 showed no significant changes. This distinct response to inflammatory stress suggests that F2CAA may modulate the inflammatory cascade differently depending on the type of stressor.

When **HaCaT** cells were **treated** with **F2CAA,** after **H_2O_2** stress, all three receptors (GPR55, CNR2, and CNR1) were significantly downregulated. This broad downregulation might indicate an attempt to reduce excessive signaling triggered by oxidative stress.

In **HDF** cells **treated** with **F2CAA,** after **H_2O_2** stress, only a slight downregulation of GPR55 was observed. This slight change suggests that F2CAA's effects on cannabinoid receptors in fibroblasts may be more pronounced in preventive (pre-treatment) rather than therapeutic (post-stress treatment) applications.

Interestingly, **HaCaT** cells treated with **F2CAA,** after **TPA** stress, significant upregulation of all three receptors was observed. This contrasts with the pre-treatment results and suggests that F2CAA may enhance the skin's responsiveness to cannabinoid signaling when applied after inflammatory stress, potentially promoting resolution and repair processes.

- **F3TAC**

In **HaCaT pre-treated** with **F3TAC,** before **H_2O_2** stress, GPR55 and CNR2 showed slight upregulation, while CNR1 was strongly upregulated. This suggests a potential protective mechanism against oxidative stress, as CNR1 and CNR2 activation is generally associated with anti-inflammatory and antioxidant effects. The upregulation of GPR55, which can promote inflammation when activated, indicates a complex modulation of the inflammatory response.

In **HDF**, **F3TAC pre-treatment,** before **H_2O_2** stress, led to slight upregulation of GPR55 and CNR1, with downregulation of CNR2. This differential response in fibroblasts highlights the cell type-specific effects of F3TAC and may indicate a tailored protective mechanism in the dermal layer.

For **HaCaT** cells **pre-treated** with **F3TAC,** before **TPA** stress, GPR55 was upregulated, CNR2 was significantly downregulated, and CNR1 showed no significant changes. This distinct response to inflammatory stress suggests that F3TAC may modulate the inflammatory cascade differently depending on the type of stressor.

When **HaCaT** were treated with **F3TAC,** after **H_2O_2** stress, CNR2 was significantly downregulated, **GPR55** showed slight upregulation, and CNR1 had no significant changes. This response suggests that F3TAC may modulate the inflammatory and regenerative pathways differently when applied after oxidative stress has occurred.

In **HDF** cells **treated** with **F3TAC,** after **H_2O_2** stress, GPR55 was downregulated, while CNR2 and CNR1 were upregulated (CNR2 strongly). This pattern indicates that F3TAC may enhance cannabinoid signaling in fibroblasts following oxidative stress, potentially promoting anti-inflammatory and regenerative responses.

Interestingly, **HaCaT** cells **treated** with **F3TAC,** after **TPA** stress, showed significant upregulation of all three receptors. This suggests that F3TAC may enhance the skin's responsiveness to cannabinoid signaling when applied after inflammatory stress, potentially promoting resolution and repair processes.
